# ADP-ribosylation of RNA in mammalian cells is mediated by TRPT1 and multiple PARPs

**DOI:** 10.1093/nar/gkac711

**Published:** 2022-08-26

**Authors:** Lisa Weixler, Karla L H Feijs, Roko Zaja

**Affiliations:** Institute of Biochemistry and Molecular Biology, RWTH Aachen University, Pauwelsstrasse 30, Aachen 52074, Germany; Institute of Biochemistry and Molecular Biology, RWTH Aachen University, Pauwelsstrasse 30, Aachen 52074, Germany; Institute of Biochemistry and Molecular Biology, RWTH Aachen University, Pauwelsstrasse 30, Aachen 52074, Germany

## Abstract

RNA function relies heavily on posttranscriptional modifications. Recently, it was shown that certain PARPs and TRPT1 can ADP-ribosylate RNA *in vitro*. Traditionally, intracellular ADP-ribosylation has been considered mainly as a protein posttranslational modification. To date, it is not clear whether RNA ADP-ribosylation occurs in cells. Here we present evidence that different RNA species are ADP-ribosylated in human cells. The modification of cellular RNA is mediated by several transferases such as TRPT1, PARP10, PARP11, PARP12 and PARP15 and is counteracted by different hydrolases including TARG1, PARG and ARH3. In addition, diverse cellular stressors can modulate the content of ADP-ribosylated RNA in cells. We next investigated potential consequences of ADP-ribosylation for RNA and found that ADPr-capped mRNA is protected against XRN1 mediated degradation but is not translated. T4 RNA ligase 1 can ligate ADPr-RNA in absence of ATP, resulting in the incorporation of an abasic site. We thus provide the first evidence of RNA ADP-ribosylation in mammalian cells and postulate potential functions of this novel RNA modification.

## INTRODUCTION

Many functions of RNA rely on a chemically diverse set of RNA modifications. To date, about 150 types of RNA modifications have been identified (1). Due to their relative abundance, rRNA and tRNA modifications are best studied. Recent advances in next generation sequencing (NGS) technologies offered insight into mRNA and long non-coding RNA (lncRNA) modifications. The most abundant internal modification of mRNA is methylation of adenosine m^6^A (2–4). Beside internal modifications, mRNA can also be modified at its 5′-end during capping. Formation of a 5′-cap on newly synthesized RNA represents the first co-transcriptional modification of nascent mRNA and occurs once the nascent transcript is 20–40 nucleotide long (5,6). The canonical 5′-cap structure of eukaryotic mRNA is represented by the N^7^-methylated guanosine that is linked to the first nucleotide of RNA via reverse 5′→5′ triphosphate linkage. The m^7^G-cap (Cap 0) is essential for initiation of translation and protects nascent mRNA form premature degradation by 5′→3′ exonuclease activity. In addition, the m^7^G-cap also serves as a scaffold for recruiting protein factors involved in pre-mRNA splicing, polyadenylation and nuclear export (7,8). It has long been presumed that RNA capping does not occur in bacteria. However, recent research showed that bacterial RNAs can carry the nucleotide-containing metabolite NAD^+^ at their 5′-end (9). NAD^+^, as well as related cofactors (FAD, DP-CoA), can be attached to the 5′-end of RNA in yeast and human cells (10). In contrast to m^7^G-caps, which are co-transcriptionally introduced to the 5′-end of RNA, NAD^+^-caps are added during transcription initiation by the RNA polymerase II through the use of NAD^+^ instead of ATP as an initiating nucleotide (11). NAD^+^-capped mRNAs overlap with canonical m^7^G-capped mRNAs and were estimated to comprise 1–6% of the total mRNA. However, the proportion of small nuclear RNAs (snRNAs) is higher in NAD^+^-capped RNA populations, suggesting certain transcript-preference in NAD^+^-capping. NAD^+^-capped RNA is not translated and is instead actively degraded (12).

ADP-ribosylation is a highly conserved covalent modification in which an ADP-ribose (ADPr) moiety is transferred from the cofactor NAD^+^ to target molecules (13). Similar to other protein post-translational modifications (PTMs), ADP-ribosylation of proteins can regulate different functions including enzymatic activity, protein-protein interactions or cellular localization (14,15). Intracellular ADP-ribosylation is catalysed by PARP enzymes that belong to the family of diphtheria-toxin like ADP-ribosyltransferase (ARTDs) (16,17). Out of 17 mammalian ARTDs, 4 are able to synthetize long poly-ADPr (PAR) chains resulting in protein poly(ADP-ribosyl)ation (PARylation). The role of protein PARylation in the DNA damage response, transcription and protein degradation is well described (18). However, the majority of PARPs transfer only a single ADPr moiety, resulting in mono(ADP-ribosyl)ation (MARylation) (19–21). ADP-ribosylation is a highly dynamic and fully reversible modification. PAR chains are efficiently degraded by poly(ADP-ribose)glycohydrolase (PARG) and ADP-ribosylhydrolase 3 (ARH3) (22–25). MARylation is reversed by two structurally unrelated groups of enzymes whose activities are selective for the ADPr-acceptor amino acid. The hydrolases ARH1 and ARH3 remove ADPr attached to arginine and serine, respectively. ADPr linked to acidic residues is removed by macrodomain-containing proteins MACROD1, MACROD2 and terminal ADP-ribose glycohydrolase (TARG1) (26–28).

Thus far, proteins have been considered to be the main targets of ADP-ribosylation. However, recent evidence suggests that also nucleic acids can be ADP-ribosylated (29,30). The first enzymes described to modify DNA were pierisin toxins that irreversibly ADP-ribosylate DNA on guanine (31), followed by reversible ADP-ribosylation on thymidine in single-stranded DNA by the bacterial toxin-antitoxin system DarT/DarG (32). Nucleic acids have only recently been identified as substrate for mammalian ARTDs *in vitro*. Contrary to the known toxins, PARP1–3 modify DNA on terminal phosphates at sites of DNA breaks (33,34). In 2019 ADP-ribosylation on the 5′-phosphate of single stranded RNA (ssRNA) was shown *in vitro*, mediated by the catalytic domains of several PARPs and reversed by several hydrolases (35). In addition to PARPs, RNA 2'-phosphotransferase (Tpt1) from bacteria and fungi as well as a Tpt1 ortholog in higher organisms, TRPT1, mediate ADP-ribosylation of the terminal 5′-phosphate of ssRNA (36). Although several studies showed ADP-ribosylation of RNA by recombinant catalytic domains of certain PARPs and TRPT1 *in vitro*, it is not known whether this *in vitro* activity has any biological relevance or if the modification occurs in human cells.

In this study, we demonstrate that RNA MARylation occurs in human cells. Different pools of cellular RNA are MARylated to varying extent after hydrolase-knockdown or overexpression of different PARPs and the level of ADP-ribosylated RNA changes as a part of stress response. Furthermore, we show that MARylation of the 5′-terminal phosphate of RNA influences its stability and blocks the translation of mRNA *in vitro* and in cells. Instead, the modified RNA can be used in a ligation reaction independent of ATP. Our findings thus add ADPr as a novel non-canonical RNA-cap to the expanding variety of functionally relevant RNA modifications, which occur in mammalian cells.

## MATERIALS AND METHODS

### Oligonucleotides

The sequences of the oligonucleotides used in this study are listed in Supplementary Table S1. All oligonucleotides used in this study that were not generated by *in vitro* transcription, were purchased from Integrated DNA Technologies. The oligonucleotides were resuspended to 50–100 μM stock solutions in 20 mM HEPES–KOH (pH 7.6), 50 mM KCl buffer or RNase free water.

### Mammalian and bacterial expression constructs

For GFP-PARP overexpression PARP10, PARP11, PARP12 and PARP15 were sub-cloned from pDONR221 to a pcDNA5/FRT/TOgw-N-mEGFP gateway vector using LR clonase mix (Thermo Fischer Scientific). The destination vector pcDNA5/FRT/TOgw-N-mEGFP was made in house using the NEBuilder® HiFi DNA Assembly Cloning Kit (New England Biolabs). MACROD1 and TARG1 were cloned to pDEST17 as described (37). The pOTB7-DXO construct was obtained as MGC cDNA clone from Dharmacon. DXO was amplified by PCR using gateway compatible primers and then cloned to pDONR221 using BP clonase (Thermo Fischer Scientific). For protein purification DXO was subcloned in pDEST17 using LR clonase mix. Human ARH1, ARH2 and TRPT1 were synthesized as a gateway compatible gBLOCKs (Integrated DNA Technologies), cloned to pDONR221 and then sub-cloned to pDEST17. Human PARG catalytic domain and ARH3 purification constructs were kind gifts from L. Lehtiö (38). All mutants were made using Q5 Site-Directed Mutagenesis Kit (New England Biolabs). All plasmids that were generated during this study will be made available through Addgene (Supplementary Table S2).

### Protein purification

Bacterial cultures were spun down at 6000 × *g* for 15 min at 4°C. Bacterial pellets were suspended in lysis buffer and cells were lysed using the Digital Sonifier 250 Cell Disruptor (Branson). Sonication settings varied depending on the expressed construct and pellet size (2–5 min at 15–20%, 30 s on, 40 s off). The cell lysates were cleared by centrifugation at 15 000 × *g* for 45 min at 4°C. Proteins were purified via affinity purification with Glutathione Agarose (Pierce) or TALON Metal Affinity Resin (Takara Bio), for GST-tagged and His-tagged constructs, respectively. Eluted protein fractions were dialysed and quality checked via SDS-PAGE (Supplementary Figure S1).

### Dephosphorylation assays

Dephosphorylation assays were performed according to the manufacturer's protocol either with Shrimp Alkaline Phosphatase rSAP or RNA 5′ Pyrophosphohydrolase RppH (New England Biolabs) to remove all phosphates or to obtain monophosphorylated RNA oligos, respectively.

### ADP-ribosylation and ADP-ribosylhydrolase assay

ADP-ribosylation assays and hydrolase assays were performed in ADPr-reaction buffer (20 mM HEPES–KOH, pH 7.6, 50 mM KCl, 5 mM MgCl_2_, 1 mM DTT, 500 μM NAD^+^, 40 U RNase inhibitor (New England Biolabs)) for 30–60 min at 37°C. In reactions with synthetic RNA oligos 1–5 μM RNA was incubated with 1–2 μM transferase or hydrolases. Proteins were digested with 20 U Proteinase K (New England Biolabs) for 20 min at RT before samples were resolved on denaturing urea-PAGE. Samples were completed with RNA loading dye (New England Biolabs), heated up for 3 min at 85°C and loaded on pre-run urea polyacrylamide gels (8 M urea, 10–15% acrylamide:bisacrylamide (19:1), 0.2% APS, 0.4% TEMED). Gels were run in TBE buffer (89 mM Tris, pH 8.0, 89 mM boric acid, 2 mM EDTA) at 7 W and stained as required with SYBR™ Gold nucleic acid gel stain (Invitrogen). If radiolabelled ^32^P-β-NAD^+^ was used for *in vitro* RNA modification or the samples were used in subsequent assays, ADP-ribosylation reactions were purified after Proteinase K treatment using the Monarch® RNA Cleanup Kit (New England Biolabs). ADP-ribosylation of the *G*Luc reporter was performed as described for the synthetic oligos, after treating the product of *in vitro* transcription (IVT) with RppH to obtain mono-phosphorylated *G*Luc mRNA. Additionally, the ADPr-*G*Luc reporter sample was treated for 1 h at 37°C with XRN-1 (New England Biolabs) to remove non-modified RNA. RNA concentrations were determined by spectrophotometric measurements (NanoDrop ND-1000 Spectrophotometer).

### Ligation assay and APE1 treatment

Ligation assays were performed using the T4 RNA ligase 1 (T4 Rnl1) or RtcB ligase (New England Biolabs). Reactions were assembled according to the manufacturer's protocols and supplemented with 7.5% PEG 8000 and 40 U RNase inhibitor T4 Rnl1 reactions were incubated at 16°C for 16 h or at 25°C for 2 h. RtcB ligase reactions were incubated for 1 h at 37°C. Apurinic/apyrimidinic Endonuclease 1 (APE1) (New England Biolabs) was used to cleave the phosphodiester backbone of double stranded DNA:RNA hybrids 5′ to abasic sites. RNA ligation products were hybridised with a reverse complementary DNA oligo by heating at 80°C for 5 min and cooling-down to RT. Hybridised samples were supplemented with 1 μl NEBuffer 4 and 5 U APE1 in a total volume of 10 μl. Reactions were incubated at 37°C for 1 h.

### *In vitro* transcription and NAD^+^ capping

*In vitro* transcribed ssRNA oligos were generated using the MEGAshortscript™ T7 Transcription Kit (Invitrogen). Reactions were assembled according to the manufacturer's protocol. For IVT product_21nt and IVT product_40nt 3 μg DNA template was used in 20 μl reaction volume and reactions were incubated at 37°C overnight. The radiolabelled NAD^+^-capped RNA oligo was generated by the addition of ^32^P-β-NAD^+^ to the standard IVT reaction of IVT product_40nt; its template sequence was designed with one exclusive thymidine at the 5′-end of the DNA to enable exclusively labelling of NAD^+^-caps. *G*Luc reporter was generated by using 1 μg of template DNA and incubation at 37°C for 4 h. *G*Luc reporter was poly(A) tailed using *E. coli* Poly(A) Polymerase (New England Biolabs). Reaction was assembled according to manufactures protocol and incubated for 30 min at 37°C. All IVT reactions were DNase treated for 30 min at 37°C, using 1 μl TURBO™ DNase (Invitrogen). RNA was purified using the Monarch® RNA Cleanup Kit (New England Biolabs).

### Cloning of *G*Luc reporter and *in vitro* translation assay

A *Gaussia* luciferase reporter construct containing a T7 promoter, a short 5′-UTR and minimal 3′-UTR was purchased as a gBLOCK from Integrated DNA technologies. The construct was amplified by PCR and used for *in vitro* transcription after column purification as described. The *in vitro* transcribed mRNA was poly(A) tailed, purified and m^7^G-capped using the *Vaccinia* Capping System (New England Biolabs) or ADP-ribosylated using human TRPT1 after dephosphorylation using RppH to produce monophosphorylated RNA suitable for ADP-ribosylation. These mRNAs were then used in *in vitro* translation assays using the PURExpress^®^*in vitro* protein synthesis kit (New England Biolabs) according to the manufacturer's protocol with 2 μg of m^7^G-capped, ADPr-capped or non-capped *G*Luc reporter mRNA. Alternatively, capped *G*Luc mRNA was co-transfected with m^7^G-capped firefly luciferase (FLuc) mRNA in HEK293T cells using Lipofectamine MessengerMAX (Invitrogen). The cell medium was collected for measurement of secreted *G*Luc at indicated time points. Cells were lysed and FLuc activity was measured in lysates to check for transfection efficiency. *G*Luc activity was measured with the GAR-2B *Gaussia* Luciferase assay (Targetingsystems). FLuc activity in cell lysates was measured using the Steady-Glo Luciferase Assay System (Promega).

### RNA isolation and detection of ADP-ribosylated RNA by slot blot

Isolation of total RNA fractions was carried out with the RNeasy Mini Kit (Qiagen) or the RNeasy Plus Mini Kit (Qiagen) according to the manufacturer's protocols. Small (≤200) and large (≥200) RNA fractions were isolated using the microRNA Purification Kit (Norgen) according to the manufacturer's protocol. Enrichment of RNA was performed with the Dynabeads™ mRNA DIRECT™ Purification Kit (Invitrogen) from the total or large RNA fraction. 200 μl oligo (dT) 25 beads were used for 100 μg of total RNA. After equilibration of the beads in lysis/binding buffer, total RNA, diluted in lysis/binding buffer, was added to the bead suspension and incubated for 5 min at RT while rotating. Beads were washed according to the manufacturer's protocol and eluted in elution buffer while shaking at 750 rpm at 80°C for 2 min.

RNA was blotted on Hybond-N^+^ membrane (Amersham) using the Slot Blot Blotting Manifold (Hoefer). The membrane was pre-wetted in 10xSSC-buffer, pH 7.0 (150 mM NaCl, 15 mM sodium citrate). Slots were rinsed with 200 μl 10× SSC-buffer before loading. 40–500 ng RNA in 200 μl SSC-buffer was applied to the membrane. After the membrane was dried for at least 30 min samples were UV-cross-linked with 120 mJ/cm^2^. For antibody staining the membrane was blocked with 5% non-fat milk in PBST (PBS with 0.05% Tween 20) for 60 min at RT and subsequently incubated with the Anti-PAR/MAR (E6F6A0, Cell Signalling Technology (CST)) in 1:7000 dilution in PBST at 4°C overnight. The HRP-conjugated Peroxidase AffiniPure Goat Anti-Rabbit IgG (H + L) secondary antibody (Jackson) was diluted 1:7,500 in 2% non-fat milk in PBST and incubated for 1 h at RT. Multiple washing steps with PBST were performed after both antibody incubations for at least 5–10 min. Chemiluminescence signals were detected using the WesternBright Chemiluminescence Substrate Quantum (Biozyme) and the detection system Azure600 (Azure Biosystems) or X-ray films (Fuji).

### Cell culture and transfection

HeLa S3 cells were kept at a humidified atmosphere at 37°C with 5% CO_2_ and were cultivated in DMEM with high glucose and GlutaMAX™ (Gibco) supplemented with 10% heat-inactivated foetal bovine serum (Gibco). Hydrolase knock-downs were performed by reverse transfection using Dharmacon siRNA smartpools or single siRNAs (Supplementary Table S3) and Lipofectamine RNAiMax (Invitrogen) as per manufacturer's instructions at a final siRNA concentration of 10 nM. Cells were seeded in six-well plates at 150 000/well. After 24–48 h cells were washed and transfected with 2 μg of plasmid DNA, using 4 μl Lipofectamine 2000 reagent mixed in Opti-MEM™ I Reduced Serum Medium (Gibco) to overexpress mEGFP-PARPs or TRPT1. Transfected cells were washed after 4–5 h and incubated another 24–48 h before collection. *G*Luc mRNA was transfected using Lipofectamine messengerMAX transfection reagent (Invitrogen). For detection of ADPr-RNA upon cellular stress, HeLa cells were treated with MG132 (16 h, 2 μM), hydrogen peroxide (3 h, 300 μM), sodium arsenite (1 h, 250 μM) and interferon alpha (16 h, 180 U/ml). Cell starvation was done by washing cells twice in PBS and incubation in EBSS, containing calcium and magnesium (Gibco), for 3 h.

### Western blot

Cell protein extractions were performed using RIPA buffer (150 mM NaCl, 1% Triton X-100, 0.5% sodium deoxycholate, 0.1% SDS, 50 mM Tris–HCl (pH 8.0)) supplemented with protease inhibitor cocktail, followed by benzonase treatment (Santa Cruz Biotechnology). Proteins were separated on 10–15% gels and blotted onto nitrocellulose. Membranes were blocked with 5% non-fat milk in TBST (TBS with 0.05% Tween 20) for 1 h at RT, primary antibodies were diluted in TBST and incubated overnight at 4°C, secondary antibodies were diluted in 5% non-fat milk in TBST and incubated for 1 h at RT. Wash steps were performed in between and after antibody incubations with TBST at RT for at least 5 min. The HRP-conjugated Peroxidase AffiniPure Goat Anti-Rabbit IgG (H + L) secondary antibody (Jackson) was diluted 1:7500 in 2% non-fat milk in PBST and incubated for 1 h at RT. Chemiluminescence signals were detected by either exposure to film or using the Azure600. A monoclonal antibody that recognises TARG1 was raised against the full-length His-tagged protein in rat (37). ARH3 was detected using a mouse monoclonal antibody (sc-374162; Santa Cruz) and for detection of PARG a rabbit monoclonal antibody was used (66564; Cell Signalling Technology). Transient overexpression of mEGFP-PARPs was detected using a goat polyclonal anti-GFP antibody (600-101-215; Rockland).

## RESULTS

### RNA is ADP-ribosylated in human cells

**Figure 1.**
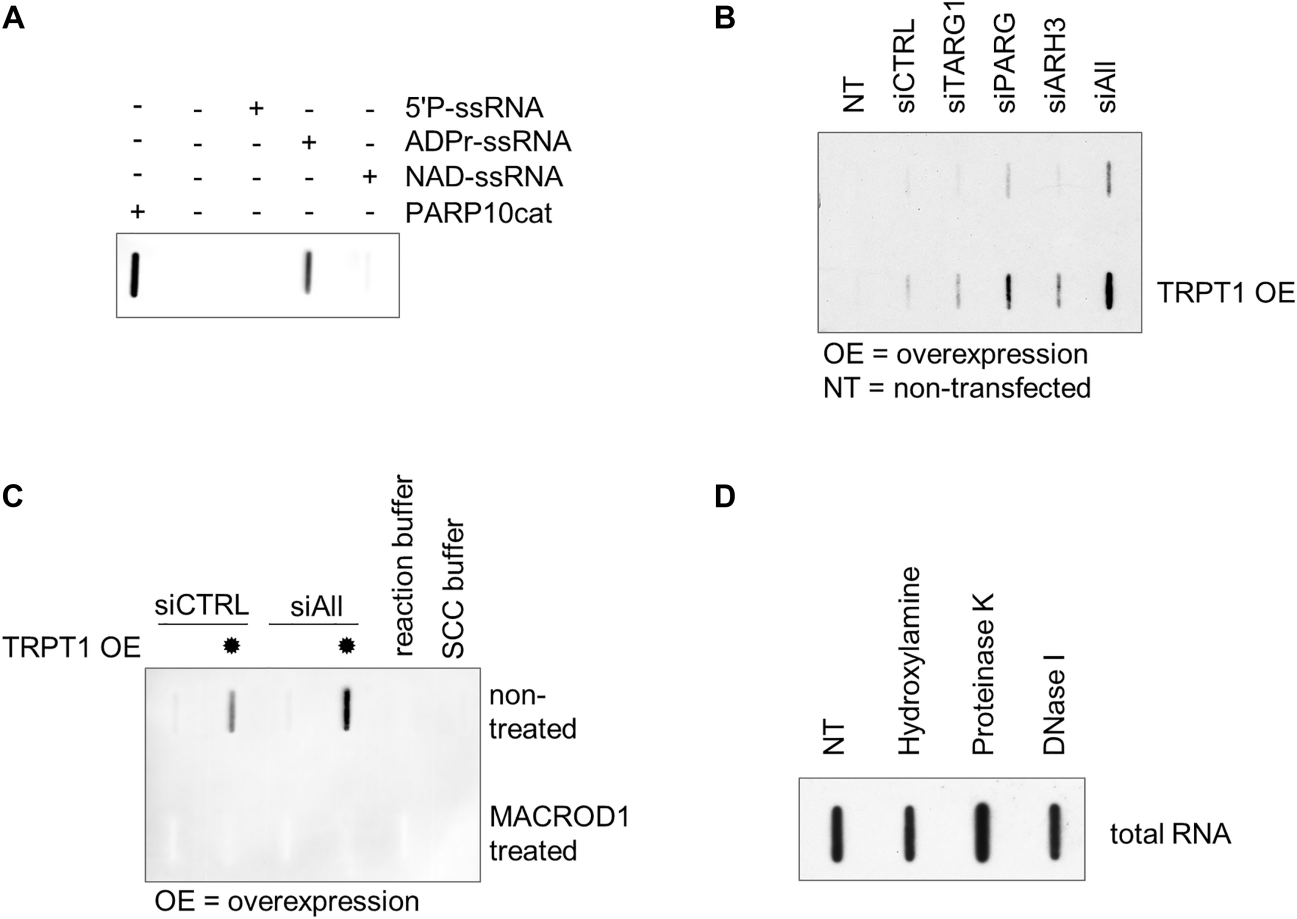
RNA is ADP-ribosylated in mammalian cells. (**A**) A synthetic 5′-phosphorylated ssRNA oligo (5′P-GUU UCG GAU CGA CGC-3′OH) was used in an ADP-ribosylation assay with TRPT1. TRPT1 was incubated with this oligo and NAD^+^ at 37°C for 30 min to generate ADPr-capped RNA. NAD^+^-capped RNA was generated co-transcriptionally during *in vitro* transcription where ATP was replaced by NAD^+^ (5′NAD-GG CCU CUC GCU CUG CUG GGU GUG CGC UUG CUU GGC UUG C-3′OH). After NAD^+^- or ADPr-capping, proteins were digested by proteinase K treatment, followed by RNA purification. Auto-modified PARP10cat was used as positive control for MARylation and 5′-phosphorylated synthetic ssRNA was used as negative control. Samples were blotted onto a positively charged nylon membrane and probed with a poly/mono ADP-ribose antibody (E6F6A0). (**B**) HeLa cells were reverse transfected with siRNA to knock-down either TARG, ARH3, PARG or a combination of all three (siAll). After 24 h, cells were transfected with a TRPT1-encoding plasmid. 48 h after transfection, total RNA was isolated, slot-blotted and probed as described in (A). Replicates are shown in Supplementary Figure S11A. (**C**) HeLa cells were prepared as in (B). Total RNA was extracted and incubated with MACROD1 at 37ºC for 30 min. RNA samples were blotted and analysed via immunoblotting as described in (A). (**D**) RNA was extracted from transferase overexpressing cells, in combination with hydrolase triple knock-down, as described in (B). 500 ng total RNA was incubated in ADPr-buffer with 1 M hydroxylamine (RT; 4 h), DNase I (37°C; 30 min) or proteinase K (37°C; 30 min). Samples were blotted and probed with the poly/mono ADP-ribose antibody (E6F6A0) as described in (A).

Two previous studies showed that short 5′-phosphorylated RNA oligos can be *in vitro* ADP-ribosylated by several human PARPs (35,36), however, to date there is no evidence that RNA is ADP-ribosylated in cells. Recently different antibodies against ADP-ribosylated proteins were developed, which provides an opportunity to study endogenous ADP-ribosylation. We systematically tested the specificity of these reagents toward ADP-ribosylated proteins as well as nucleic acids (39). Based on these results, we chose the commercially available poly/mono-ADP Ribose (E6F6A) antibody from Cell Signalling Technology for the detection of endogenous ADP-ribosylated RNA. To demonstrate the specificity of this antibody we generated ADPr-capped, as well as the structurally related NAD^+^-capped RNA, and blotted these together with non-capped RNA and the auto-modified catalytic domain of PARP10 (PARP10cat). While non-capped RNA and NAD^+^-capped RNA are not recognised by the antibody, both auto-modified PARP10cat as well as purified ADPr-capped RNA were detected (Figure 1A**)**. This confirms the specificity of the used antibody for ADPr-modification independent of the substrate-backbone and enables the usage of this reagent for the detection of ADPr-RNA.

Although the catalytic domains of several PARPs can ADP-ribosylate RNA *in vitro* (35), TRPT1 is the only human full-length transferase that ADP-ribosylates RNA *in vitro* (35,36), although modification of RNA with ADP-ribose has not been observed in cells yet. We hypothesised that the potential modification of RNA with ADPr is highly dynamic and efficiently reversed by cellular hydrolases, which would explain the lack of evidence of modification in cells so far. To detect cellular ADPr-RNA we therefore overexpressed TRPT1 and in addition silenced the hydrolases PARG, TARG1 and ARH3, either in single or in triple knock-downs to slow down the turnover of ADPr-RNA (Supplementary Figure S2). Although MACROD1 and MACROD2 are also active on ADPr-RNA *in vitro*, they are not expected to interfere in this setting due to MACROD1’s mitochondrial localization and the low expression level of MACROD2 (37). From the transfected cells we isolated and slot blotted total RNA to detect ADP-ribosylated RNA. For RNA from cells without hydrolase knock-down or TRPT1 overexpression the signal is not detectable, while TRPT1 overexpression or hydrolase single knock-down alone induced a weak ADPr-signal (Figure 1B). Single hydrolase-silencing in combination with TRPT1 overexpression increases the signal intensity in all samples, while the combination of TRPT1 overexpression and hydrolase triple knock-down leads to the strongest increase of ADPr-RNA in cells (Figure 1B). The used anti-ADPr antibody also detects adenylylated RNA (App-RNA) (39,40). App-RNA is resistant to MACROD1 treatment *in vitro* (Supplementary Figure S3). To verify that the observed signal is indeed ADP-ribosylation we incubated total RNA samples with the recombinant ADP-ribosylhydrolase MACROD1. After MACROD1 treatment of the cellular RNA, the signal disappeared, corroborating that the signal results from an ADP-ribosylated substrate (Figure 1C). Treatment of cellular ADPr-RNA with either hydroxylamine, DNase I or proteinase K did not lead to a loss of the signal, confirming the previously observed resistance of the ADPr-phospho-linkage to hydroxylamine (41) and excluding the possibility that the observed signal is derived from contaminant ADP-ribosylated DNA or protein (Figure 1D). These results provide evidence that ADP-ribosylation of RNA takes place in mammalian cells and represents a dynamic RNA modification whose turnover is influenced by TRPT1 overexpression and hydrolase activity.

### Human TRPT1 ADP-ribosylates 5′-terminal, monophosphorylated purines

**Figure 2.**
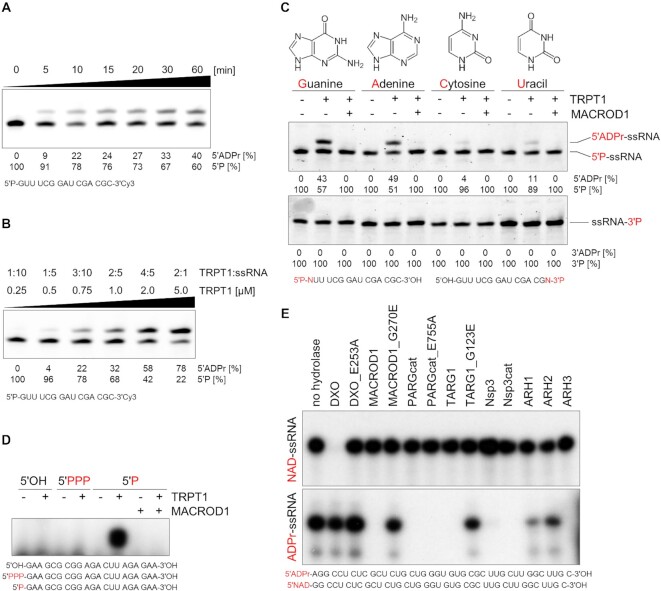
Human TRPT1 ADP-ribosylates specific 5′-monophosphorylated RNA substrates *in vitro*. (**A**) 2.5 μM of a synthetic 5′-phosphorylated, 3′-Cy3 labelled ssRNA oligo was incubated with 1 μM TRPT1 at 37ºC for the indicated incubation periods. Samples were proteinase K treated and reactions were separated via urea-PAGE. In-gel fluorescence of the Cy3-lable was detected. Ratios of modified and non-modified RNA are indicated below the lanes, expressed as percent of total Cy3-labelled RNA. (**B**) 2.5 μM of a synthetic 5′-phosphorylated, 3′-Cy3 labelled ssRNA oligo were incubated with the indicated amounts of TRPT1 at 37°C for 30 min. Samples were proteinase K treated and reactions were separated via urea-PAGE. In-gel fluorescence of the Cy3-label was detected. Ratios of modified and non-modified RNA are indicated below the lanes, expressed as percent of total Cy3-labelled RNA. (**C**) 5′-phosphorylated or 3′-phosphorylated synthetic ssRNA oligos with different 5′- or 3′-terminal nucleotides (indicated as N) were incubated with TRPT1 at 37ºC for 30 min followed by incubation with MACROD1 at 37ºC for 30 min. Proteins were degraded by proteinase K and reactions resolved via urea-PAGE. The gel was stained with SYBR gold nucleic acid gel stain to visualise the RNA. Ratios of modified and non-modified RNA are indicated below the lanes, expressed as percent of total Cy3-labelled RNA. (**D**) 5′-triphosphorylated ssRNA was transcribed *in vitro*. The generated oligo was dephosphorylated or monophosphorylated by rSAP or RppH, respectively. The oligos with different 5′-phosphorylation states (non-phosphorylated; triphosphorylated; monophosphorylated) were used in ADP-ribosylation assays with TRPT1 and ^32^P-labelled NAD^+^. After proteinase K treatment samples were resolved via urea-PAGE, followed by autoradiography detection. (**E**) *In vitro* transcribed ADPr-capped ssRNA was prepared with labelled NAD^+^ as described in (D). NAD^+^-capped RNA was generated using *in vitro* transcription in presence of ^32^P-labelled NAD^+^. Modified RNA substrates were incubated with the indicated hydrolases at 37°C for 30 min. Samples were proteinase K treated and resolved via urea-PAGE, followed by autoradiography detection. DXO-E253A, TARG1-G123E, MACROD1-G270E are catalytically inactive mutants. PARGcat_E755A = poly(ADP-ribose) binding mutant, cat = catalytic domain.

In our experiments, the transferase TRPT1 has neither auto-modification activity nor ADP-ribosylation activity on protein substrates (Supplementary Figure S4A). However, TRPT1 can bind RNA molecules (Supplementary Figure S4B). As shown, overexpression of TRPT1 in HeLa cells leads to an increase in RNA ADP-ribosylation (Figure 1B and C). This makes TRPT1 the only known RNA-specific ADP-ribosyltransferase. Two previous studies tested the ADP-ribosylation activity of human TRPT1 towards RNA, but reported contradictory results (35,36). To assess the activity of human TRPT1 on ssRNA, we incubated TRPT1 with a ssRNA oligo in a time course as well as in a protein titration assay. RNA ADP-ribosylation leads to a reduced mobility of the RNA oligo and is visible as a slower migrating band on the denaturing urea gel. Under the given conditions, 1 μM TRPT1 modifies 40% of 2.5 μM ssRNA after 60 min (Figure 2A), while the ADPr-RNA modification levels are increased by raising the ratio of protein to RNA (Figure 2B). It is worth mentioning that the discrepant studies used RNA oligos with different 5′-terminal nucleotides, namely cytidine (36) and guanosine (35). To test whether human TRPT1 exhibits base-specificity, we incubated TRPT1 with RNA oligos that only differ in their 5′-terminal nucleotide. TRPT1 shows a strong preference for purines (guanine/adenosine) while pyrimidines (cytosine/uracil) are only weakly modified (Figure 2C, upper panel). Hydrolase MACROD1 reversed modification of all four ADP-ribosylated RNA oligos (Figure 2C, upper panel). These findings indicate that TRPT1 exhibits 5′-terminal base specificity and thereby provides an explanation of the different activities observed in previous studies. To test whether PARPs also exhibit base preferences, we incubated different oligos with recombinant PARP10, PARP10cat, PARP11 and PARP11cat. The catalytic domain of PARP11 can weakly modify all nucleobases to the same extent, whereas PARP10cat modifies preferentially pyrimidines (Supplementary Figure S5A). Under the given conditions, there was no detectable activity for the full-length PARP10 and PARP11. *Clostridium thermocellum* and *Aeropyrum pernix* TRPT1 homologs are active on 3′-phoshorylated RNA oligos (42), while no 3′-activity was shown for human TRPT1 (35). To test whether the apparent lack of 3′-activity of human TRPT1 is also caused by base specificity, TRPT1 was incubated with 3′-monophosphorylated oligos that differ in their 3′-terminal nucleotide. No activity could be detected for 3′-phosphorylated RNA regardless of the 3′-terminal nucleotide (Figure 2C, lower panel), confirming the specificity of human TRPT1 toward 5′-phosphorylated ssRNA oligos.

Newly transcribed mRNA is 5′-triphosphorylated before capping and in mammalian cells 5′-monophosphorylated RNA is efficiently degraded by 5′→3′ exoribonuclease XRN1/2 (43). ADP-ribosylation of RNA *in vitro* occurs on a terminal monophosphate (35); however it is not known whether di- or triphosphorylated RNA can be ADP-ribosylated. To test whether TRPT1 can modify RNA with multiple 5′-phosphates, we generated non-phosphorylated, mono- and triphosphorylated oligos*. In vitro* transcribed, triphosphorylated RNA was enzymatically treated with RNA 5′-pyrophosphohydrolase (RppH) or shrimp alkaline phosphatase (rSAP), to obtain monophosphorylated and non-phosphorylated oligos, respectively (Supplementary Figure S5B). These oligos were incubated with TRPT1 and ^32^P-labelled NAD^+^. While 5′-monophosphorylated RNA was extensively modified by TRPT1, we did not observe activity on either non-phosphorylated or triphosphorylated substrates (Figure 2D).

Not only ADPr can be attached to the 5′-end of RNA, but also NAD^+^ can function as cap (44). Contrary to ADPr and the canonical m^7^G cap, RNA is capped with NAD^+^ co-transcriptionally by RNA polymerase II. The NAD^+^-cap is removed by the exoribonuclease DXO (12). To test the specificity of diverse ADP-ribosylhydrolases on ADPr- and NAD^+^-capped RNA, we generated capped ssRNA oligos with ^32^P-β-NAD^+^ that are modified with either ADPr or NAD^+^. The activities of purified ADP-hydrolases were first confirmed on protein substrates (39) and next RNA substrates were incubated with either DXO or specific ADP-ribosylhydrolases. The NAD^+^-cap was removed by DXO but not by the catalytically inactive DXO mutant E253A; however, none of the ADP-ribosylhydrolases showed activity towards NAD^+^-capped RNA (Figure 2E, upper panel). In contrast, the ADPr-cap was efficiently removed by hydrolases that are capable of cleaving *O*-glycosidic bonds. The previously reported catalytically inactive mutants of hydrolases that are not able to reverse ADP-ribosylation of proteins showed no activity on any RNA substrate. Notable exception is the E755A mutant of PARGcat which is not active on protein PARylation (23) but efficiently hydrolysed ADPr-RNA (Figure 2E, lower panel). The decrease of signal after ARH1 and ARH2 treatment is not due to hydrolase activity, but caused by RNA degradation due to contaminant in the protein preparations (Supplementary Figure S6). Together, these results confirm that TRPT1 exclusively ADP-ribosylates 5′-monophosphorylated RNA with preference towards purines. This modification results in a non-canonical RNA cap that is reversible by a set of ADP-ribosylhydrolases.

### Capping of mRNA with ADPr prevents degradation and translation

**Figure 3.**
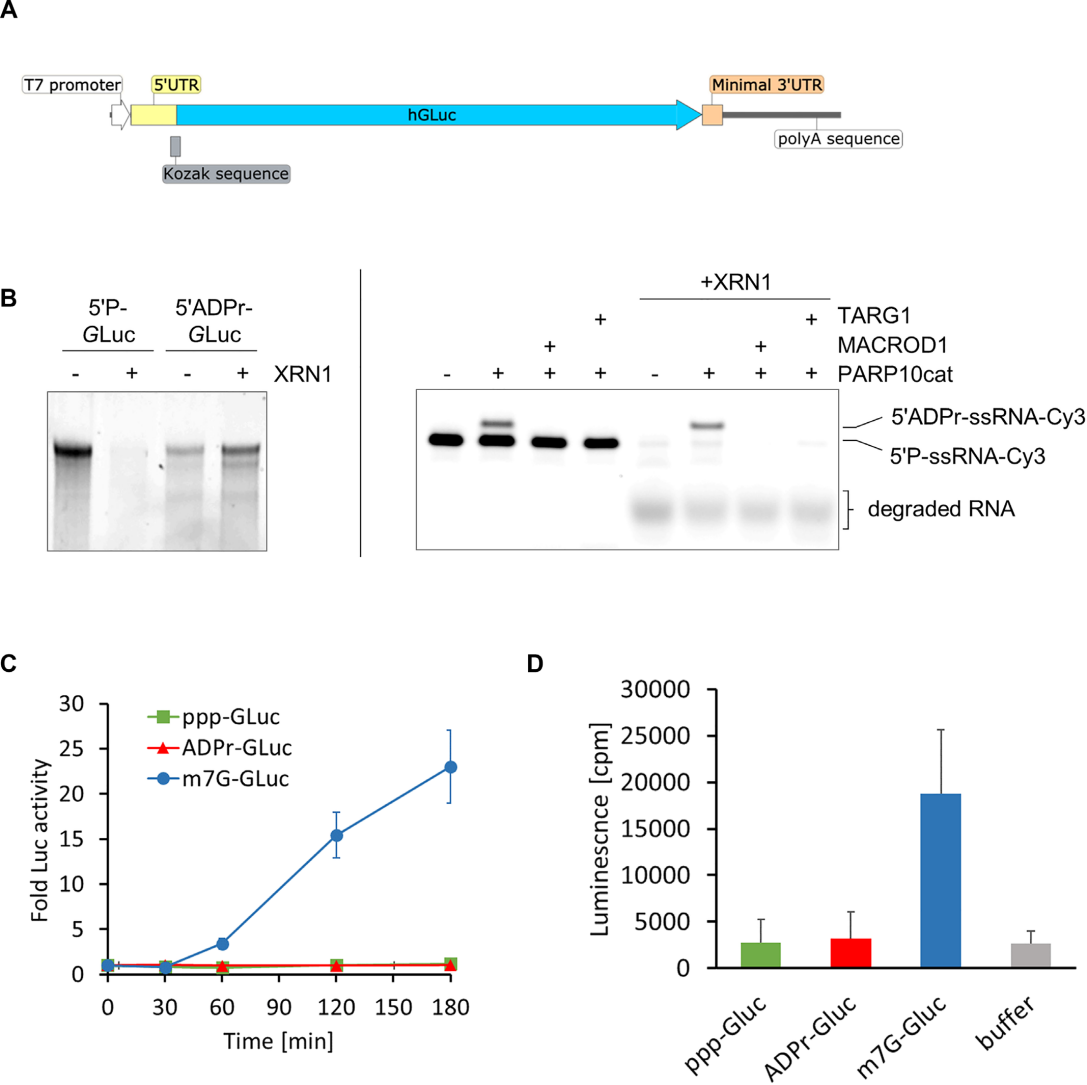
ADP-ribosylated RNA is protected from degradation but cannot be translated. (**A**) Schematic representation of *Gaussia* Luciferase reporter (*G*Luc reporter). *G*Luc reporter RNA was produced by *in vitro* transcription, followed by poly-A tailing to prevent 3′-degradation. The T7-promotor and Kozak-sequence were introduced to enable transcription and translation, respectively. (**B**) *In vitro* transcribed and poly-A tailed *G*Luc reporter RNA was monophosphorylated with RppH, purified and ADP-ribosylated with TRPT1 (left panel). In parallel, a synthetic 5′-phosphorylated and 3′-Cy3 labelled ssRNA oligo was modified with TRPT1 and treated with MACROD1 (right panel). Monophosphorylated or ADPr-capped *G*Luc reporter RNA (left panel), or synthetic RNA (right panel), were incubated with the 5′-monophosphate dependent 5′→3′ exoribonuclease XRN1 at 37°C for 1 h. After proteinase K treatment, reactions were resolved via urea-PAGE. RNA was visualised with SYBR gold nucleic acid gel stain (left panel) or by the detection of in-gel fluorescence of the Cy3-label (right panel). (**C**) ADPr-capped *G*Luc reporter RNA was produced as described in (B) followed by removal of non-modified species by XRN1 mediated degradation and purification. m^7^G-capped RNA was generated using the *Vaccinia* Capping System after *in vitro* transcription and poly-A tailing of the *G*Luc reporter RNA. 0.5 μg differently capped or triphosphorylated *G*Luc reporter RNAs were transfected into HeLa cells (96-well plates) using MessengerMax. Cells were incubated for different periods to allow translation of the reporter mRNA and secretion of luciferase. The medium was harvested and analysed using luminescence-assays. (**D**) 2 μg of each *G*Luc reporter RNA that was generated in (C) were utilised in an *in vitro* translation assay using the PURExpress^®^*in vitro* protein synthesis kit. Measured luminescence reflects the rate of translation of *G*Luc constructs after 2 h. In (C) and (D), mean and standard deviation of three independent experiments are shown.

RNA-caps have important regulatory functions. The main function of the canonical m^7^G-cap is the protection of mRNA from degradation and to recruit translation factors, whereas the NAD^+^-cap blocks efficient translation and leads to degradation of the modified RNA (12). To test the *in vitro* susceptibility of ADPr-capped RNA to degradation, we generated a *Gaussia* Luciferase reporter (*G*Luc-reporter) mRNA (Figure 3A and Supplementary Table S1) and incubated this with the 5′-monophosphate specific 5′→3′ exoribonuclease XRN1. ADP-ribosylation protects the *G*Luc-reporter from degradation, whereas monophosphorylated, non-capped *G*Luc-reporter mRNA was completely degraded (Figure 3B, left panel). The same effect was observed for synthetic ssRNA oligos that are ADP-ribosylated (Figure 3B, right panel). Next, we tested whether ADPr-capped mRNA can be translated. We transfected purified triphosphorylated, ADPr-capped or m^7^G-capped *G*Luc*-*reporter mRNA into HeLa cells and measured the secretion of *Gaussia* luciferase. The m^7^G-mRNA transfected cells show a linear increase in luminescence, indicating efficient translation of the *G*Luc-reporter, while no increase in luciferase signal could be measured for ADPr-capped or non-capped *G*Luc-reporter (Figure 3C). The lack of translation of ADPr-mRNA could be the consequence of fast degradation after de-capping by intracellular hydrolases. To test this, we measured translation efficiency of the ADP-ribosylated *G*Luc reporter mRNA in an *in vitro* translation assay. The m^7^G-capped *G*Luc reporter was translated, indicated by an average ∼7 fold increase of the luciferase signal in comparison to the buffer sample. However, the luciferase signal of ADPr-capped mRNA corresponded to the translation level of the triphosphorylated RNA or buffer only control, which served as additional negative controls (Figure 3D). These results demonstrate that ADPr-capped mRNA is not translated, but is protected against 5′→3′ nuclease activity.

### RNA can be ligated through ADP-ribose in the absence of ATP

**Figure 4.**
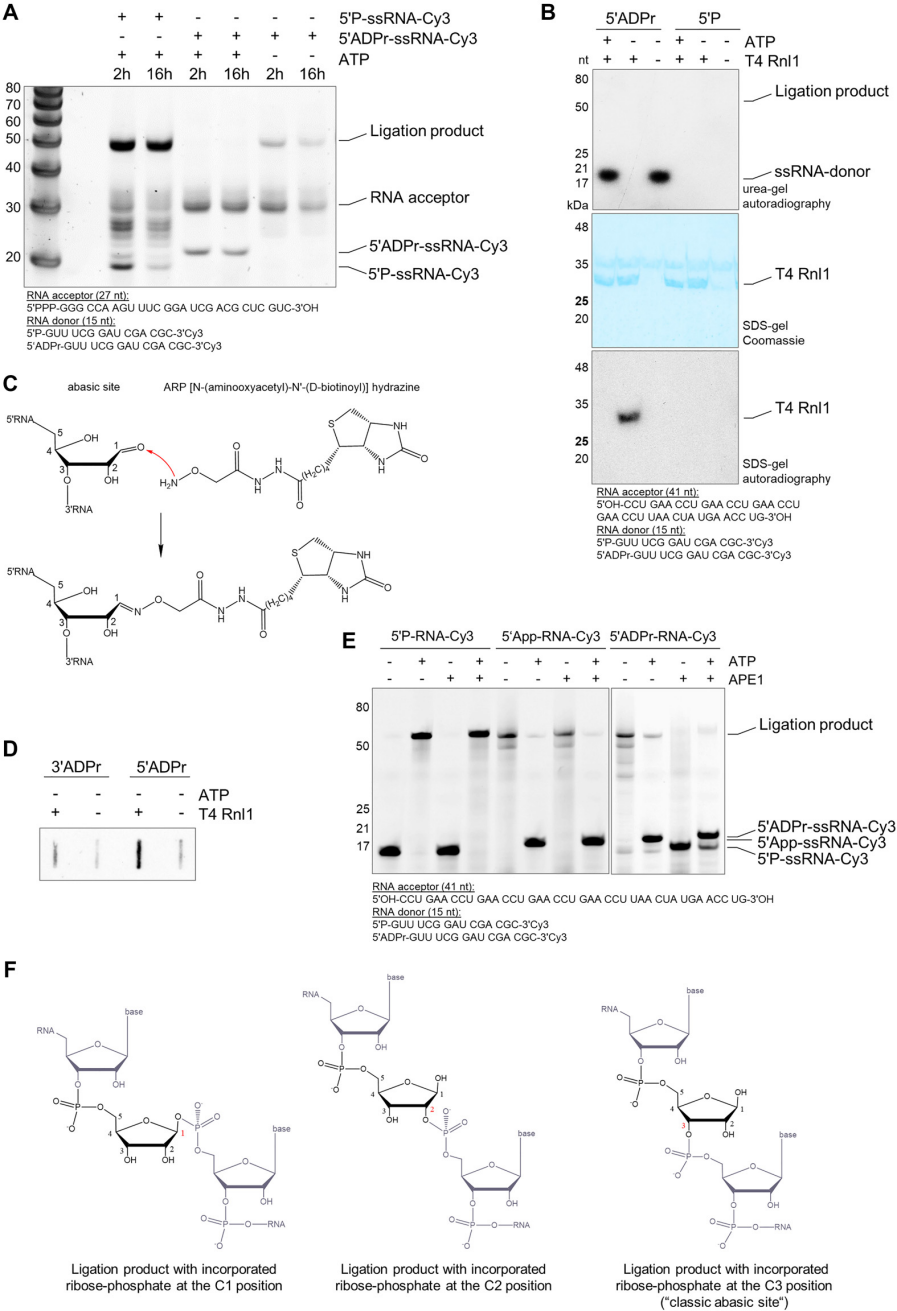
ADP-ribosylated RNA is ligated by T4 RNA ligase 1 in absence of ATP. (**A**) A 15 nt 5′-phosphorylated and 3′-Cy3 labelled ssRNA oligo was incubated with TRPT1 at 37ºC for 30 min. Samples were XRN-1 and subsequently proteinase K treated and purified. A 27 nt triphosphorylated oligo was generated as RNA-acceptor using IVT. Approximately 1 μM ADPr-capped oligo and 2.5 μM 5′-phosphorylated oligo were ligated to 4.5 μM RNA acceptor using 20 U T4 RNA ligase 1 with or without 1 mM ATP. Reactions were incubated at 25°C for 2 h or at 16°C for 16 h, proteinase K treated, resolved via urea-PAGE and stained with SYBR gold nucleic acid gel stain. (**B**) A 15 nt 5′-p-ssRNA oligo was incubated with TRPT1 and ^32^P-NAD^+^, followed by purification. 0.5 μM 41 nt non-phosphorylated RNA acceptor and 0.1 μM RNA donor were ligated with 10 U T4 Rnl1 in presence or absence of 1 mM ATP. After incubation at 16°C for 16 h samples were divided. One aliquot was proteinase K treated and resolved via urea-PAGE. The second aliquot was resolved via SDS-PAGE and Coomassie stained. For both autoradiography was detected. (**C**) The exposed aldehyde group of oxidised abasic sites can complex with [N-(aminooxyacetyl)-N'-(D-biotinoyl)] hydrazine (ARP). (**D**) 5′-phosphorylated and 3′-Cy3 labelled, or 3′-phosphorylated and 5′-Cy3 labelled ssRNA oligos were incubated with TRPT1 or PARP10cat respectively, at 37ºC for 30 min and purified. 0.5 μM of TRPT1-treated RNA (∼70% modification rate; ∼0.35 μM 5′-ADPr-RNA) or ∼0.5 μM of PARP10cat-treated RNA (∼65% modification rate; ∼0.33 μM 3′-ADPr-RNA) RNA oligos were ligated with 2 μM acceptor RNA oligo and 10 U T4 Rnl1 ligase in absence of ATP. After incubation at 16°C for 16 h, T4 Rnl1 was inactivated at 65°C for 15 min. Ligation reactions were incubated with ARP at 37°C for 1 h, cross-linked with PFA and purified. Samples were blotted and probed for abasic sites using Streptavidin-HRP. (**E**) A 15 nt 5′-phosphorylated and 3′-Cy3 labelled ssRNA oligo was ADP-ribosylated by TRPT1. After purification, 0.8 μM of 5′-monophosphorylated RNA or 5′-ADPr-RNA were ligated with 2 μM RNA acceptor (41 nt) and 20 U T4 Rnl1 at 16°C for 16 h. Next T4 Rnl1 was inactivated at 65°C for 15 min. A reverse complementary DNA oligo was annealed to the ligation products. DNA:RNA hybrids were treated with 5 U purinic/apyrimidinic endonuclease 1 (APE1) at 37°C for 1 h. Following proteinase K treatment, reactions were resolved via urea-PAGE and in-gel fluorescence was detected. SYBR gold nucleic acid gel stain dyed gel is shown in Supplementary Figure S6C. (**F**) Hypothetically possible incorporation variants of the ribose-5′-phosphate after ligation of ADPr-RNA, where the variant on the right has been shown to occur experimentally. Grey = RNA backbone; black = ribose-phosphate.

ATP-dependent RNA ligases join 3′-hydroxyl and 5′-phosphorylated RNA termini via three reaction steps: (i) the ligase reacts with ATP to form a covalent ligase-(lysyl-Nζ)–AMP intermediate with concomitant release of pyrophosphate; (ii) AMP is transferred from ligase-adenylate to the 5′-phosphorylated RNA to form an adenylylated RNA intermediate (App-RNA); 3) the 3′-hydroxyl of the RNA acceptor attacks the adenylylated RNA to join RNA ends through a phosphodiester 3′→5′ bond (Supplementary Figure S7) (45–48). 5′-ADP-ribosylated RNA resembles the App-RNA intermediate in ligation step 2 but contains an additional ribose-phosphate proximal to the RNA. Therefore, we tested if ADPr-RNA can serve as activated App-RNA-donor mimic in a ligation reaction with the ATP-dependent T4 RNA ligase 1 (T4 Rnl1) from *E. coli*. We generated 5′-ADPr-RNA that is 3′-Cy3 labelled to prevent circularisation. In a reaction with 1 mM ATP, T4 Rnl1 ligates 5′-monophosphorylated RNA but not 5′-ADPr-RNA (Figure 4A). In contrast, the 5′-ADPr-oligo is ligated by T4 Rnl1 in the absence of ATP (Figure 4A). This corresponds to the behaviour of App-RNA that likewise can only be ligated in absence of ATP (Supplementary Figure S8A). The apoenzyme of T4 Rnl1 can thus ligate an ADPr-capped or adenylylated RNA oligo; this ligation is inhibited when T4 Rnl1 is already activated by ATP. To test whether ADPr is retained within the ligation product, we ADP-ribosylated RNA with radiolabelled β-NAD^+^ (adenylate^32^P) and used the oligo as RNA-donor in a ligation assay with T4 Rnl1. The radiolabel of the ADP-ribosylated RNA-donor disappeared upon ligation but did not reappear at the size of the expected ligation product. We did not measure potential release of AMP, however, we could detect AMP bound to the ligase (Figure 4B). This indicates that the AMP moiety of ADPr is not integrated into the ligation product but does not clarify whether the remaining ribose-5′-phosphate is incorporated. Ribose-5′-phosphates correspond to RNA abasic sites, which were shown to be present in different organisms (49). Abasic sites can have a closed or open configuration of the ribose-ring (Supplementary Figure S8B). To probe whether ADPr-mediated ligation leads to an abasic site, we used a biotinylated hydroxylamine [(*N*-(aminooxyacetyl)-*N*'-(d-biotinoyl)] hydrazine (ARP) which reacts with the aldehyde groups that are exposed at oxidised abasic sites (Figure 4C). Using ARP, we detected an enriched signal for abasic sites in the 5′-ADPr-ligation sample compared to control reactions, indicating that ligation through ADPr leads to an unconventional reaction product, which might be abasic sites (Figure 4D; Supplementary Figure S8C). To further substantiate this finding, we used the human apurinic/apyrimidinic endonuclease 1 (APE1), which specifically cleaves the phosphodiester backbone 5′ to abasic sites of double stranded (ds) RNA, dsDNA or DNA:RNA hybrids (50–52). Synthetic 3′-Cy3 labelled 5′-monophosphorylated RNA, adenylylated RNA or ADP-ribosylated RNA were used as RNA-donors in a ligation assay with T4 Rnl1. The ligation products were hybridised with reverse complementary ssDNA to generated DNA:RNA hybrids that were treated with APE1. Both controls, 5′-monophosphorylated RNA and App-RNA, show no processing upon APE1 treatment (Figure 4E; Supplementary Figure S8D). The ligation product of ADPr-capped RNA is cleaved, indicated by disappearance of the ligation product as well as by the appearance of an RNA fragment at the approximate size of the 5′-monophosphorylated RNA donor. This confirms that a ribose-5′-phosphate is integrated into the RNA upon ADPr-RNA ligation. It was previously proposed that ADPr is linked via the C1' to 5′-phosphorylated RNA (36). However, the presence of an abasic site detected by ARP points to an incorporated ribose-5′-phosphate that is linked via the C3′ (Figure 4F). Whether this linkage is a result of a rearrangement of the ribose-5′-phosphate bond to take the energetically favourable or most stable state before or during ligation remains to be elucidated. We next tested whether the orthologue of the only known human RNA ligase RTCB (HSPC117) is able to ligate ADPr-RNA (53). Contrary to T4 Rnl1, *E. coli* RtcB is GTP dependent and joins RNA 3′-phosphate and 5′-hydroxyl ends through the polynucleotide-(3)pp(5)G intermediate in a three-step reaction (54,55). Therefore, we generated 3′-ADPr-RNA in addition to the 5′-ADPr-RNA and tested both in a ligation assay with RtcB in presence and absence of GTP. RtcB ligates the 3′-phosphate in the positive control but shows no activity on either 3′- or 5′-ADPr-RNA (Supplementary Figure S8E). In summary, 5′-ADP-ribosylated RNA can be ligated in the absence of ATP by T4 Rnl1, resulting in a ligation product that contains an incorporated ribose-5′-phosphate.

### Different RNA pools can be ADP-ribosylated in mammalian cells

**Figure 5.**
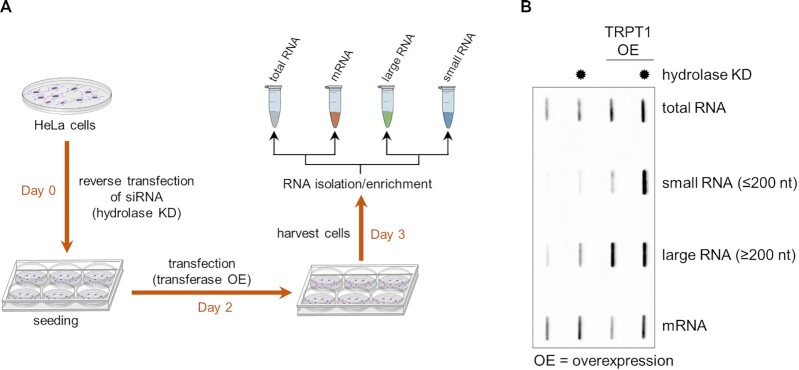
TRPT1 overexpression leads to ADP-ribosylation of different cellular RNA pools**. (A**) Scheme of the sample preparation to extract ADP-ribosylated RNA pools from cells. HeLa cells were reverse transfected in 6-well plates with siRNA to knock-down TARG1, PARG and ARH3. 48 hours after siRNA transfection, plasmids overexpressing transferases were transfected. 24–48 h later cells were harvested and total, large, and small RNA pools were isolated from cell extracts using a neutral buffer. mRNA was enriched from the total RNA fraction using oligo(dt) beads. (**B**) RNA samples from transiently TRPT1 overexpressing and wild-type cells in combination with hydrolase knock-downs were prepared as described in (A) and blotted onto a positively changed nylon membrane. ADP-ribosylation of the blotted RNAs was detected using a poly/mono ADP-ribose antibody (E6F6A0).

Our initial experiments indicate that the level of ADP-ribosylated RNA in cells is low due to reversal by hydrolases and increases upon TRPT1 overexpression (Figure 1B and C). We therefore prepared different pools of RNA (total RNA, large RNA, small RNA and mRNA) from TRPT1 overexpressing cells in combination with siRNA mediated triple knock-down of PARG, TARG and ARH3 (Figure 5A). All RNA pools were isolated under neutral conditions, as the ADPr-RNA bond is labile in acidic environment (Supplementary Figure S9). The quality of all RNA samples was analysed prior to further analysis (Supplementary Figure S10A and S10B). Total RNA is mostly comprised of ribosomal RNA (rRNA) (85%), followed by transfer RNA (tRNA) (10–12%). The mRNA together with various small and long noncoding RNAs make up only about 2–5% of the total RNA. The small RNA pool, with RNAs up to 200 nt, contains mainly tRNAs and 5S rRNA but is also enriched in microRNAs (miRNAs). The large RNA pool represents all RNAs over 200 nt in length. The RNA pools were isolated from control and hydrolase triple knock-down cells in combination with TRPT1 overexpression and slot blotted to allow detection of ADP-ribosylation. The different RNA fractions contain molecules with varying length, leading to differences in the molarity of the loaded pools, despite loading of the same mass of RNA. The resulting signal intensities can thus be compared within one RNA pool, but not between the pools. TRPT1 overexpression in cells leads to enhanced modification of diverse RNA pools, which is further increased upon hydrolase knockdown for small RNAs (Figure 5B). The mRNA fraction is not influenced by TRPT1 overexpression, however, here the signal increases upon hydrolase knockdown, hinting that other cellular enzymes are modifying mRNA although the turnover is high. These findings indicate that ADP-ribosylation of RNA is not a generic response to for example overexpression stress, but that different enzymes ADP-ribosylate different RNA species. Mammalian cells thus contain pools of RNA that are ADP-ribosylated.

### RNA ADP-ribosylation levels are modulated by PARP overexpression and cellular stress

**Figure 6.**
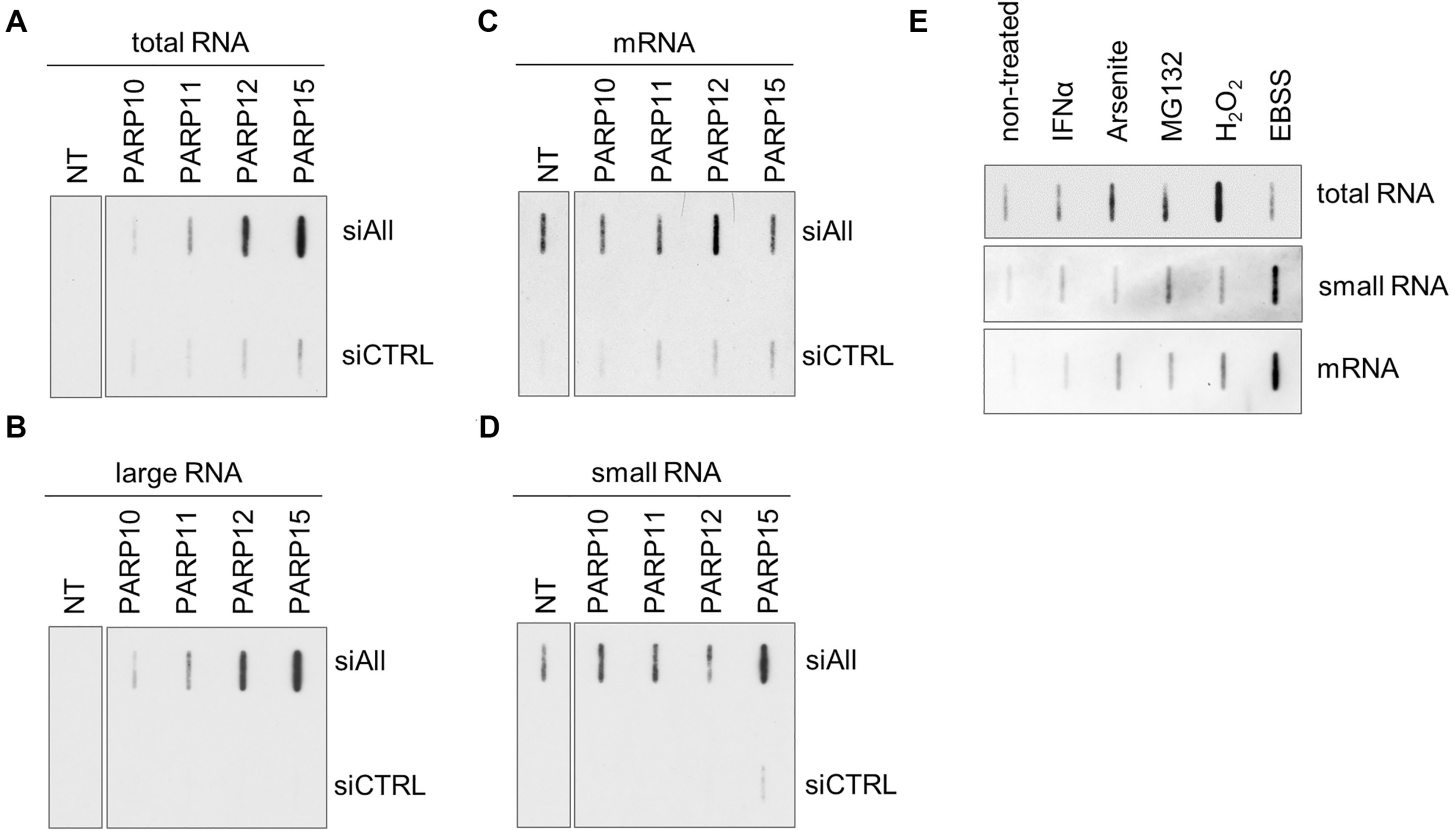
Overexpression of several PARPs as well as different stresses lead to ADP-ribosylation of diverse cellular RNA species. (**A–D**) HeLa cells were reverse transfected in six-well plates with siRNA to knock-down TARG1, PARG and ARH3. 48 hours after siRNA transfection, plasmids overexpressing indicated transferases were transfected. The different RNA pools were extracted using neutral conditions and blotted onto a positively charged nylon membrane. ADP-ribosylation signals were probed by poly/mono ADP-ribose antibody (E6F6A0). Non-cut blots and replicates are shown in Supplementary Figure S11B and S11C. (**E**) HeLa cells were seeded in 6-well dishes and treated with indicated stimuli: IFNα (16h; 180U/ml); arsenite (1 h; 250 μM); MG132; (16 h; 2 μM); H_2_O_2_ (3 h; 300 μM); EBSS medium (3 h). Isolated RNA pools were blotted onto a positively charged nylon membrane and probed with the poly/mono ADP-ribose antibody (E6F6A0).

Some PARPs, like PARP1 and PARP2, dramatically change their activity upon interaction with other proteins such as HPF1 (56,57). Despite the apparent lack of *in vitro* activity of full-length PARPs on RNA, we tested whether PARP10, PARP11, PARP12 and PARP15 are important for the modification of RNA in cells, as cellular co-factors may allow them to modify RNA. These PARPs were chosen because their catalytic domains were able to modify RNA molecules with ADPr *in vitro* or evidence in literature exists about their involvement in RNA metabolism (58). The mEGFP-tagged PARPs were transiently transfected in combination with hydrolase knock-down prior to RNA isolation (Figure 5A). In the total and large RNA pools of cells without PARP overexpression, the ADPr-signal is only visible after long exposures whereas the mRNA and small RNA pool show ADP-ribosylation upon hydrolase knockdown (Figure 6A–D). The overexpression of PARPs leads to an increase of ADPr-RNA levels in specific RNA pools and hydrolase knock-down leads to further enhancement of the ADPr-RNA signal. This indicates that multiple PARPs can modify RNA in cells, which is counteracted by ADP-ribosylhydrolases, although further studies are needed to define precisely which RNA pools are affected by which transferases and hydrolases. The varying modification rates resulting from overexpression of different PARPs, point to different RNA targets and diverse roles of PARPs in RNA modification.

PARP1–3 are activated by DNA damage, whereas the mono-ARTs PARP10, PARP11 and PARP12 are upregulated during viral infection leading to translational shut-down (59,60). To test the effect of diverse stresses on the level of ADP-ribosylated RNA, we treated HeLa cells with different stressors that ultimately lead to translational inhibition through different mechanisms prior to isolation of total RNA (Figure 6E). The pro-inflammatory IFNα was used to simulate defence against viral infection. Sodium arsenite targets the translation initiation factor 4E that binds to mRNA caps and was used to inhibit translation. The proteasome inhibitor MG132 was used to inhibit proteasomal degradation, starvation was induced by incubating cells in EBSS. H_2_O_2_ was included as known activator for PARP1–3 and to inhibit translation caused by oxidative stress. Differences are visible in response to the different stimuli, with different RNA pools affected by different stressors. This highlights that the cellular content of ADPr-RNA changes in response to diverse stresses. Future work needs to dissect which pathways are influenced and which enzymes regulate ADP-ribosylation of RNA upon stress.

## DISCUSSION

Previous work has shown that the catalytic domains of PARP10, PARP11 and PARP15 can modify RNA *in vitro* (35), while activity for the full-length proteins has not yet been convincingly demonstrated. In line with these results, we were not able to modify RNA using full length PARP10 and PARP11. It is possible that for the tested transferases, the correct conditions or substrates that would allow the modification of nucleic acids are not discovered yet. Two recent studies (35,36) that reported contradictory results regarding activity of human TRPT1, used RNA oligos starting with different nucleotides. This prompted us to test TRPT1 and PARP10cat base specificity. Contrary to the catalytic domain of PARP10, which preferentially modifies RNA oligos starting with cytidine and uridine, TRPT1 almost exclusively modifies 5′-phosphates of guanosine or adenosine. Thus, we demonstrated that TRPT1 modifies RNA oligonucleotides starting with purines but not with pyrimidines, explaining the discrepancy in the previous studies. However, full length PARP10 and PARP11 did not show transferase activity under the tested conditions regardless of the starting nucleotide of the RNA substrate.

In cells PTMs, interacting proteins as well as the cellular environment can potentially change enzyme activity and specificity. Our initial attempts to detect ADPr-RNA in cellular RNA prepared using TRIzol or phenol/chloroform were unsuccessful. Considering the properties of the *O*-glycosidic phosphoester bond in ADPr-RNA we tested its stability in an acidic environment and found that the ADPr-RNA linkage is acid labile. This finding precludes the possibility to prepare the cellular RNA by standard acid phenol-based extraction methods and explains the lack of signal in the initial attempts to detect ADPr-RNA in cells. Accordingly, we used a column-based approach to prepare cellular RNA and were able to detect the modification. In contrast to the apparent lack of *in vitro* activity, we observed ADP-ribosylation in different RNA pools upon overexpression of PARP10, PARP11, PARP12 and PARP15 in HeLa cells, indicating that they can modify RNA in the right cellular environment. Combined knock-down of hydrolases TARG1, PARG and ARH3, resulted in an increased signal for ADPr-RNA in cellular total RNA, suggesting that ADP-ribosylation of RNA occurs in mammalian cells and is reversed by cellular hydrolases. This shows that the ADPr modification is indeed highly dynamic.

Several PARPs including PARP12 and PARP15 localize to stress granules (SGs) and influence their dynamics (61–63). We have previously shown that also the hydrolase TARG1 can localise to SGs (37). SGs are dynamic structures that quickly dissolve upon subsiding of the stressor. Consequently, SGs were postulated to be sites of temporary mRNA storage that can be further processed after release of cellular stress during the recovery phase (64). This would be in line with our observation that ADPr-capped RNA is resistant to 5′→3′ exoribonuclease activity by XRN1 but is not translated. To be translated, ADPr-mRNA has to be de-ADP-ribosylated and then re-capped with a canonical cap. Although mRNA capping normally takes place in the nucleus, cytoplasmic (re)capping of mRNA has been shown to be important during stress recovery (65,66). Based on these data we hypothesize that mRNA can be modified by certain cellular PARPs localising to SGs, where mRNA would then be protected from degradation until relief of the stress. This would enable cells to quickly respond once the stress abates without wasting energy and time by the need to first go through transcription. The observed increase in ADPr-RNA after exposure to inhibitors of cellular translation that also induce SG formation, could provide additional support for this hypothesis. Further research is needed to establish the presence and role of ADPr-RNA in SGs.

Protein ADP-ribosylation is a highly dynamic PTM, which is efficiently removed by several hydrolases. We confirmed that RNA ADP-ribosylation can be reversed by the macrodomain containing hydrolases TARG1, MACROD1, Nsp3 and PARG as well as ARH3. In cells, this could serve to release ADPr-RNAs for re-capping and subsequent translation. TARG1 and MACROD1 Gly to Glu mutants block the access of ADPr to the catalytic site (26,28) and expectedly abolish hydrolase activity. PARG degrades the PAR chains through hydrolysis of the ribose-ribose *O*-glycosidic bonds and is not able to cleave the ester bond between the last ADPr moiety and the acceptor protein, however, it can reverse RNA ADP-ribosylation (35). PARG Glu 755 is important for PAR binding and its orientation, while Glu 756 is important for acid base catalysis of PAR. Either of these mutations render the protein inactive toward PAR (23). However, a PARGcat Glu 755 to Ala mutant was active toward ADPr-RNA indicating that the mode of PAR binding could be substantially different from ADPr-RNA binding. Further structural and biochemical studies are needed to define the mode of binding and hydrolysis of ADPr-RNA by PARG.

We observed that overexpression of TRPT1 in combination with hydrolase knock-down increases the cellular ADP-ribosylation signal in the pool of small RNAs. This RNA pool contains mostly tRNA and 5S rRNA. Despite several attempts, we were not able to detect transferase activity of TRPT1 on protein substrates. The lack of activity towards protein substrates makes human TRPT1 a *bona fide* RNA-specific ADP-ribosyltransferase that modifies a distinct set of RNA molecules. The fungal homolog of TRPT1, Tpt1, was discovered as a phosphotransferase that is essential for proper fungal tRNA splicing (67). TRPT1/Tpt1 is evolutionary highly conserved in bacteria, archaea and metazoa although their tRNA processing is different from that of fungi and does not result in a 2'-phosphate junction. A recent study identified 2'-phosphouridine at position 47 of tRNA from thermophilic archaea as a widespread tRNA modification, which can be reversed by Kpta/Tpt1. This raises the question whether a similar modification can exist in metazoa (68). However, Trpt1 is not essential for viability and its genetic ablation in *E. coli* or mice has no phenotypic consequences (69,70). Knock-out of Tpt1 in yeast is lethal due to its essential role in tRNA ligation. Following the excision of introns, the resulting exons are joined together by ligases leaving a 3′→5′ phosphodiester splice junction with a 2'-phosphate, which is removed by Tpt1 to generate mature tRNA (71). Important to note is that the lethality of the *tpt1* knockout in yeast is fully rescued by expressing ‘prespliced,’ intronless RNA, although cells were hypersensitive to sublethal concentrations of protein translation inhibitors and heat shock (72). Interestingly, the 2'-phosphouridine tRNA modification increases tRNA thermal stability under extreme growth temperatures in thermophilic bacteria and archea (68). Extrapolating from these findings, human TRPT1 might have additional functions in cellular stress response. Whether this additional function is mediated through its novel RNA specific ADP-ribosyltransferase activity remains to be investigated. The next open question concerns the consequence of ADPr for small RNA species, where it could potentially play a role in ligation. Gapped *in vitro* ADP-ribosylated dsDNA can be ligated with incorporation of an abasic site, indicating that DNA ligases can use ADPr as donor of AMP for DNA ligation (73). We showed that the ATP dependent bacteriophage T4 Rnl1 can ligate ADP-ribosylated RNA in absence of ATP, leading to a ligation product with an incorporated ribose-5′-phosphate. We used an aldehyde-reactive probe that reacts with the C1' exposed aldehyde of oxidised abasic sites (49) and confirmed the presence of an abasic site with the activity of APE1. This means that although the ribose of ADPr is initially linked through ribose C1' to RNA (36) a later chemical rearrangement results in a final product where ADP-ribose is linked to RNA through either its C2' or more likely C3′ atom. It is not clear whether this chemical rearrangement takes place immediately after ADP-ribosylation, during or after the ligation reaction. In summary, we hypothesise that ATP independent ligation of ADPr-RNA by T4 Rnl1 can lead to the formation of a ‘standard’ abasic site. RNA abasic sites are frequently present in cells and could have regulatory roles (49). Besides ADPr-capping leading to storage of mRNA for later usage, ADPr-capped RNAs could thus also serve as ligation substrate, although it is not clear yet which ligase would mediate this in human cells. The only known RNA ligase in human cells RTCB (HSPC117) is essential for the ligation of tRNAs and of mRNA in the unfolded protein response (53,74–76). It catalyses the GTP and Mn(II)-dependent joining of either 2'-, 3′-cyclic phosphate or 3′-phosphate termini to 5′-hydroxyl termini (53,54,77,78). Human RTCB ligase co-evolved with archease, a small acidic protein that expands the cofactor specificity of RTCB and enables the efficient use of ATP beside GTP (79,80). Contrary to the observations for T4 Rnl1, *E. coli* RtcB was not able to ligate ADPr-RNA (neither 5′- nor 3′-ADP-ribosylated RNA) under the tested conditions. However, *E. coli* RtcB is not able to use ATP for ligation and is strictly dependent on GTP, potentially explaining the lack of activity toward ADPr-RNA. It will be interesting to test whether human RTCB is able to use ADPr-RNA as a substrate in the presence of archease and whether ligation of RNA through ADPr plays a role in mammalian cells.

Our study has clear limitations: although we have optimised the purification and detection of ADP-ribosylated RNA from mammalian cells, the downstream readout is only semi-quantitative. Slot-blotting with an anti-ADPr antibody has allowed us to demonstrate that the modification exists in cells and is dynamic, however, we cannot confidently conclude which enzymes modify preferentially which RNA species. We furthermore sometimes observe an increase in signal upon control siRNA transfection, which implies that sample preparation and analysis need to be very careful to avoid inducing cellular stress and might thus further hint that ADPr-RNA is upregulated during cellular stress. More quantitative methods need to be developed for future analysis of the contribution of single enzymes to the ADPr-capping of RNA, ideally combined with chemical genetics approaches, knock-downs of individual enzymes and protein-RNA interaction studies to confidently identify the target RNAs of individual PARPs. Finally, our results indicate that ADPr-RNA can be ligated by a bacteriophage T4 RNA ligase, however, we have not identified the human ligase. This needs to be further investigated: which human ligase performs this reaction? Studying the mechanism via which the abasic site arises will also help to clarify whether the detected C3′-linked ligation product is the only reaction product or whether there might be an equilibrium between C1’ and C3′-linked products.

In summary, we provide the first evidence of the presence of ADP-ribosylated RNA in cells and moreover show that different pools of cellular RNA can be modulated by different ADP-ribosyltransferases and hydrolases. ADPr acts as novel mRNA cap that does not support translation but increases the stability of modified RNA by blocking degradation by exonuclease XRN-1. In addition, we observed that ADPr-RNA has a highly dynamic nature in cells with a fast turnover rate, at least partially mediated by ADP-ribosylhydrolases. Finally, ADP-ribosylated RNA can be ligated in an ATP-independent manner, resulting in a non-canonical ligation product with an abasic site. These data provide the basis for future research focused on the identification of ADPr-capped RNAs, on deciphering the role of ADPr as a novel RNA modification in cells as well as pinpointing the unique roles of the diverse ADP-ribosyltransferases and hydrolases.

## DATA AVAILABILITY

All experimental data is available in the main text or in the supplements. Expression plasmids generated will be available from Addgene. Cell lines are available on reasonable request from the authors.

## Supplementary Material

gkac711_Supplemental_File
