# Supplementary material for: ADP-ribosylation of RNA in mammalian cells is mediated by TRPT1 and multiple PARPs
Source: Nucleic Acids Res. 2022 Aug 26;50(16):9426–41. doi: 10.1093/nar/gkac711 (PMC9458441; doi:10.1093/nar/gkac711)
Supplement: gkac711_Supplemental_File [file gkac711_supplemental_file.pdf]

Supplementary Data

ADP-ribosylation of RNA in mammalian cells is mediated by TRPT1 and multiple PARPs

Lisa Weixler[1], Karla LH Feijs[1,*] and Roko Zaja[1,*]

[1] Institute of Biochemistry and Molecular Biology, RWTH Aachen University, Aachen, 52074, Pauwelsstrasse 30 Aachen, Germany

*To whom correspondence should be addressed. Tel: +492418037944; Fax: +492418082427. Email: rzaja@ukaachen.de. Correspondence may also be addressed to Karla LH Feijs. Tel: +492418080692; Fax: +492418082427. Email: kfeijs@ukaachen.de.

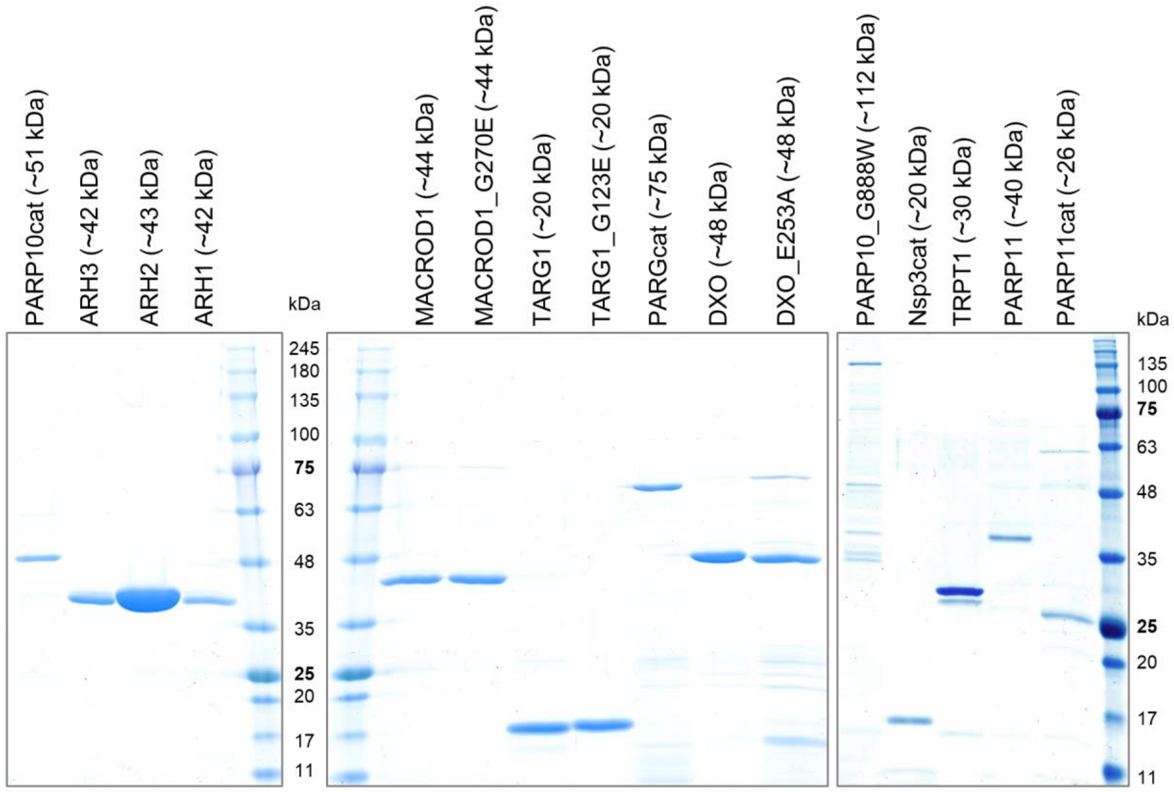

Supplementary Figure S1. Recombinant expressed and purified proteins used in this study. Proteins were recombinant expressed in *E. coli.* Expression conditions and buffer compositions were adjusted to the properties of each protein construct. Dialysed proteins were resolved via SDS-PAGE and stained with Coomassie.

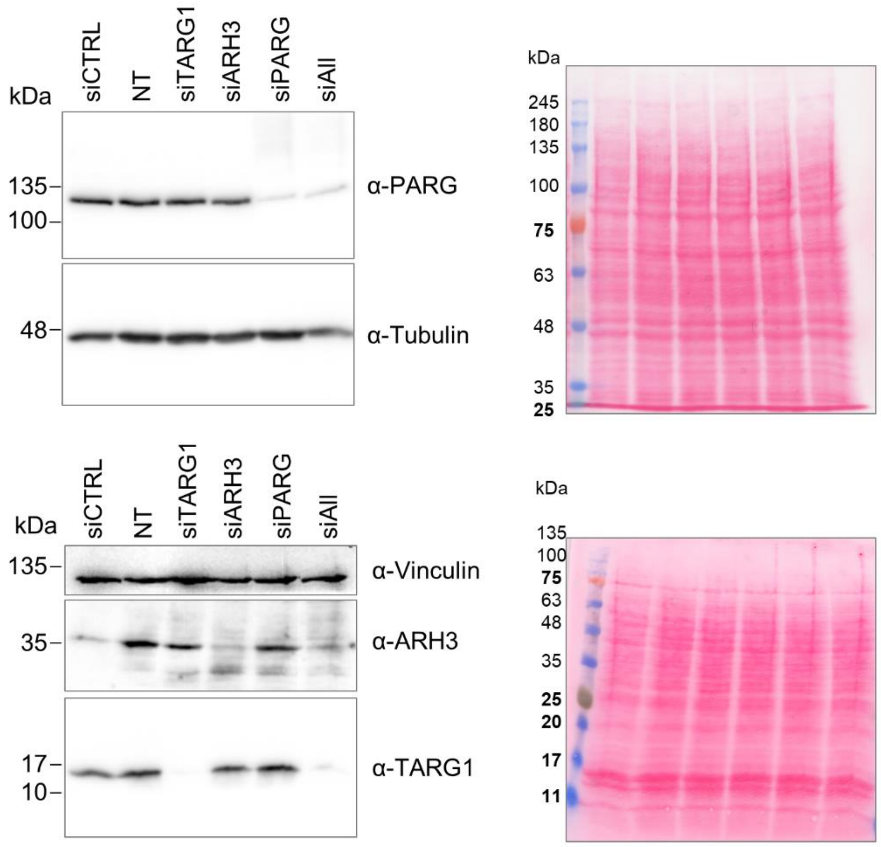

Supplementary Figure S2. siRNA transfection leads to knockdown of TARG1, PARG and ARH3. HeLa cells were reverse transfected with siRNA to knock-down TARG1, PARG and ARH3. 48 hours later cells were lysed in RIPA buffer and analysed using western blot. Antibodies used are anti-PARG (CST), anti-ARH3 (Santa Cruz) and anti-TARG1 (home-made). Vinculin and tubulin were used as loading controls. Ponceau staining of the blot is shown on the right.

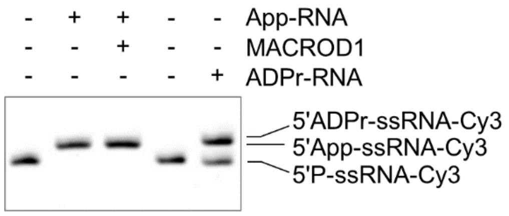

Supplementary Figure S3. MACROD1 does not reverse adenylylation of RNA.
5'-phosphorylated and 3'-Cy3 labelled synthetic ssRNA oligo (5'P-GUU UCG GAU CGA CGC-Cy3) was ADP-ribosylated with TRPT1 at 37ºC for 30 min or adenylylated using 100 pmol Mth RNA ligase at 65°C for 1 h (5'-DNA Adenylation Kit (NEB)), followed by proteinase K treatment and purification. Reactions were resolved via urea-PAGE and in-gel fluorescence was detected to visualize the shifts that result due to different modifications. The shift caused by adenylylation is not reversed by MACROD1 (37°C, 30 min).

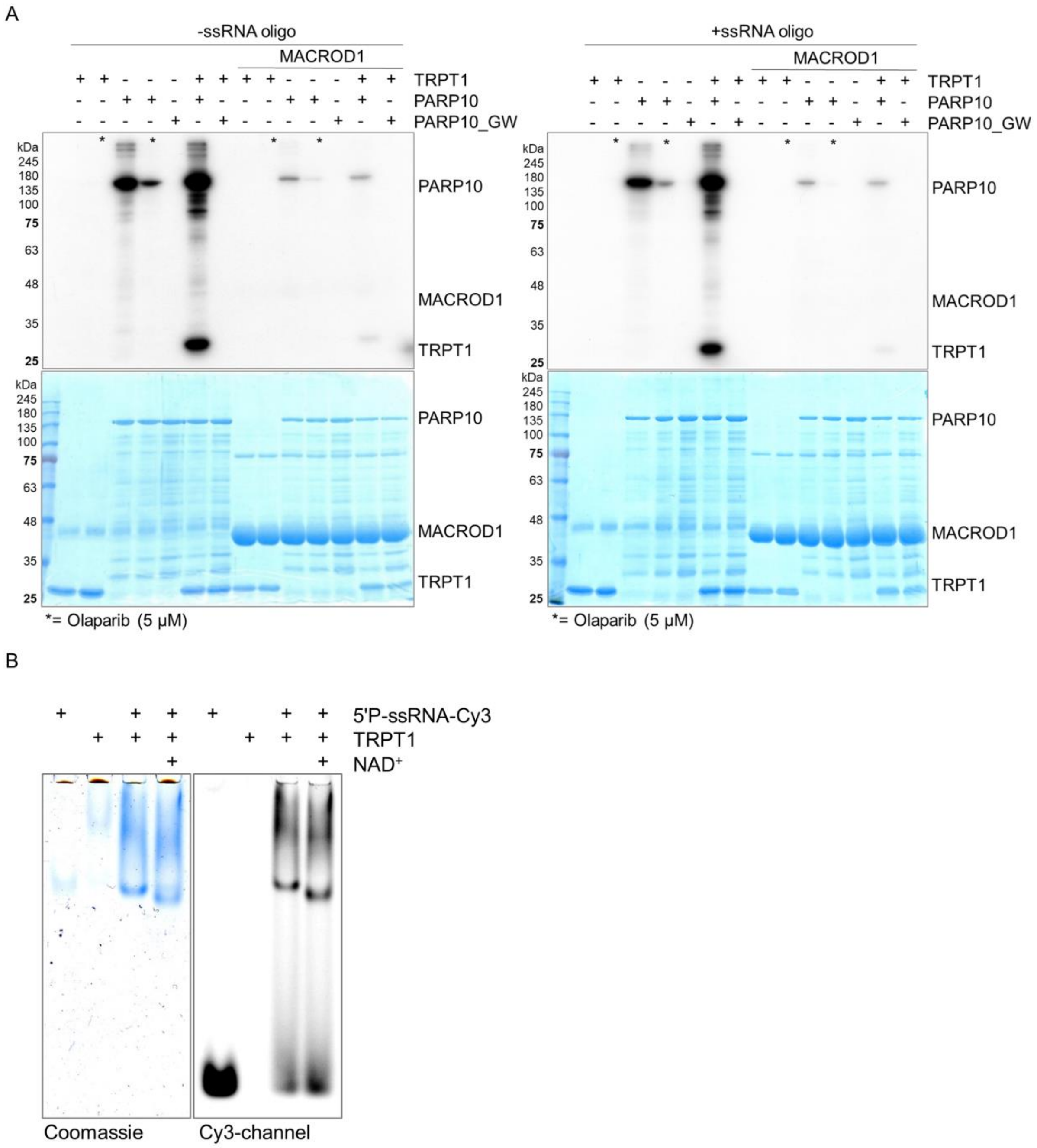

Supplementary Figure S4. TRPT1 has no activity on proteins but binds RNA and NAD$^+$.
(A) TRPT1 was incubated with PARP10 or the inactive mutant PARP10-G888W and ^{32}P-NAD$^+$ at 37ºC for 30 min. The PARP inhibitor olaparib was used to inhibit PARP10. The reactions were subsequently incubated with MACROD1 at 37ºC for 30 min. Samples were resolved via SDS-PAGE and stained with Coomassie, followed by auto-radiography detection. **(B)** 2 µM Cy3-labelled ssRNA oligo (5'P-GUU UCG GAU CGA CGC-Cy3) was incubated with 2 µM TRPT1 and 0.5 µM NAD$^+$ in 20 µL binding buffer (10 mM Tris pH 7.5; 1 mM EDTA; 100 mM KCl; 100 µM DTT, 5% glycerol; 10 µg/mL BSA) at RT for 30 min. A 5% native gel (Tris/Bis: 38:2) was pre-run at 4°C for 30 min at 100 V in TAE buffer (40 mM Tris pH 8.0; 20 mM acetic acid; 1 mM EDTA) prior to loading of the samples. Gel was run at 4°C with 100 V for 75 min. In-gel fluorescence of the Cy3-label was detected (right panel) and proteins were stained with Coomassie (left panel). Disappearance of the oligo and appearance of the protein band in the Cy3 channel in presence of TRPT1 indicates RNA binding. NAD$^+$ interaction is indicated by a shift of the protein band.

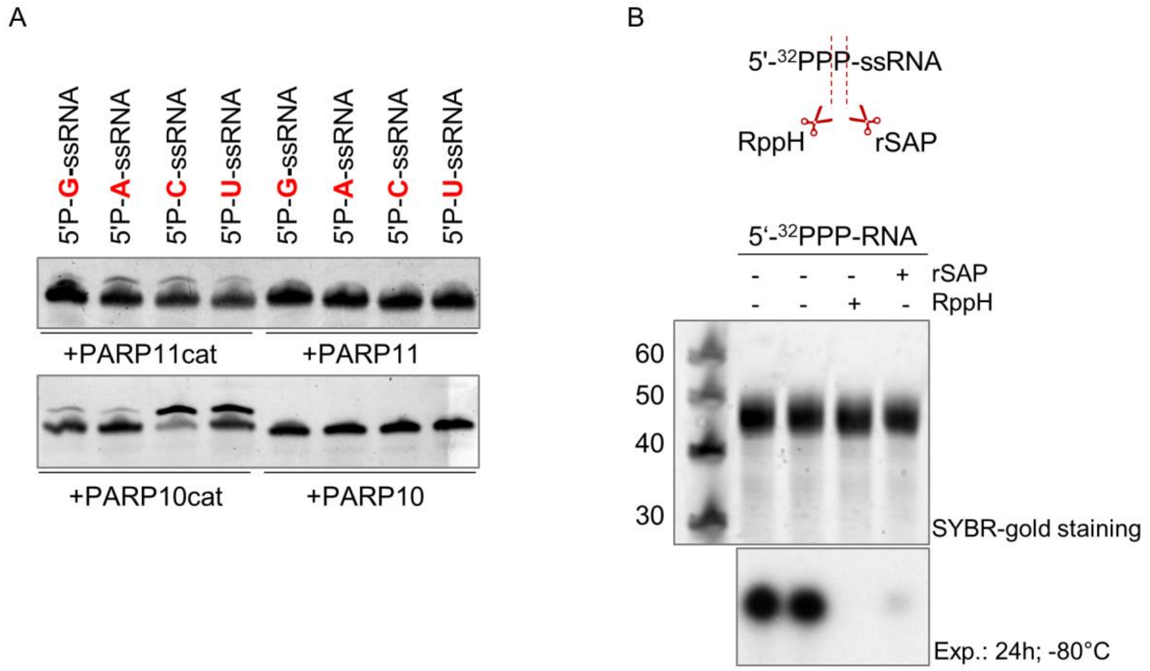

Supplementary Figure S5. Biochemical characterisation of the RNA ADP-ribosylation reaction.
(A) 2.5 µM 5'-phosphorylated ssRNA oligos with four different 5'-NTPs (5'P-NUU UCG GAU CGA CGC-Cy3) were incubated with 1 µM PARP10, PARP11 or their catalytic domains (PARP10cat; PARP11cat) and NAD^+. To visualise RNA, gel was stained with SYBR gold nucleic acid gel stain.
(B) A ssRNA oligo with a single adenine as first 5′-nucleotide (5'PPP-AGG CCU CUC GCU CUG CUG GGU GUG CGC UUG CUU GGC UUG C-OH) was generated via *in vitro* transcription (IVT) in presence of [γ-^{32}P]ATP. The IVT product is triphosphorylated and radioactive marked at the distal phosphate. The oligo was incubated with rSAP to generate non-phosphorylated RNA and with RppH to obtain monophosphorylated RNA. To visualise RNA, the gel was stained with SYBR gold nucleic acid gel stain. Dephosphorylation status was verified by a reduced autoradiography signal.

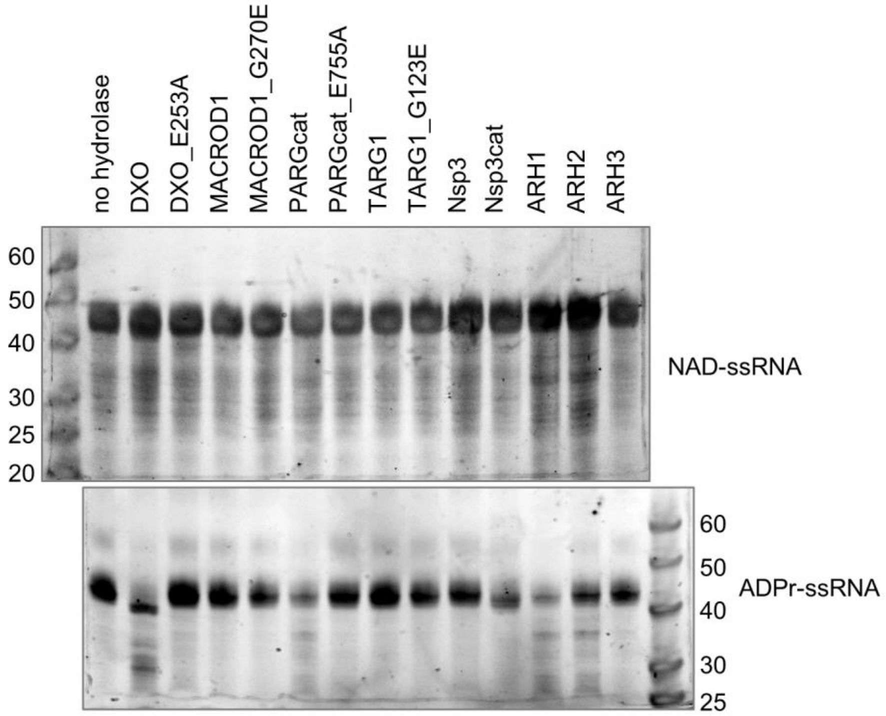

Supplementary Figure S6. ADP-ribosylation of RNA is reversed by ADP-ribosylhydrolases but not by DXO.
SYBR gold nucleic acid gel stain dyed gel belonging to the autoradiography signal shown in Figure 2E. DXO-E253A, TRAG1-G123E, MACROD1-G270E = catalytically inactive mutants, PARGcat_E755A = poly(ADP-ribose) binding mutant, cat = catalytic domain.

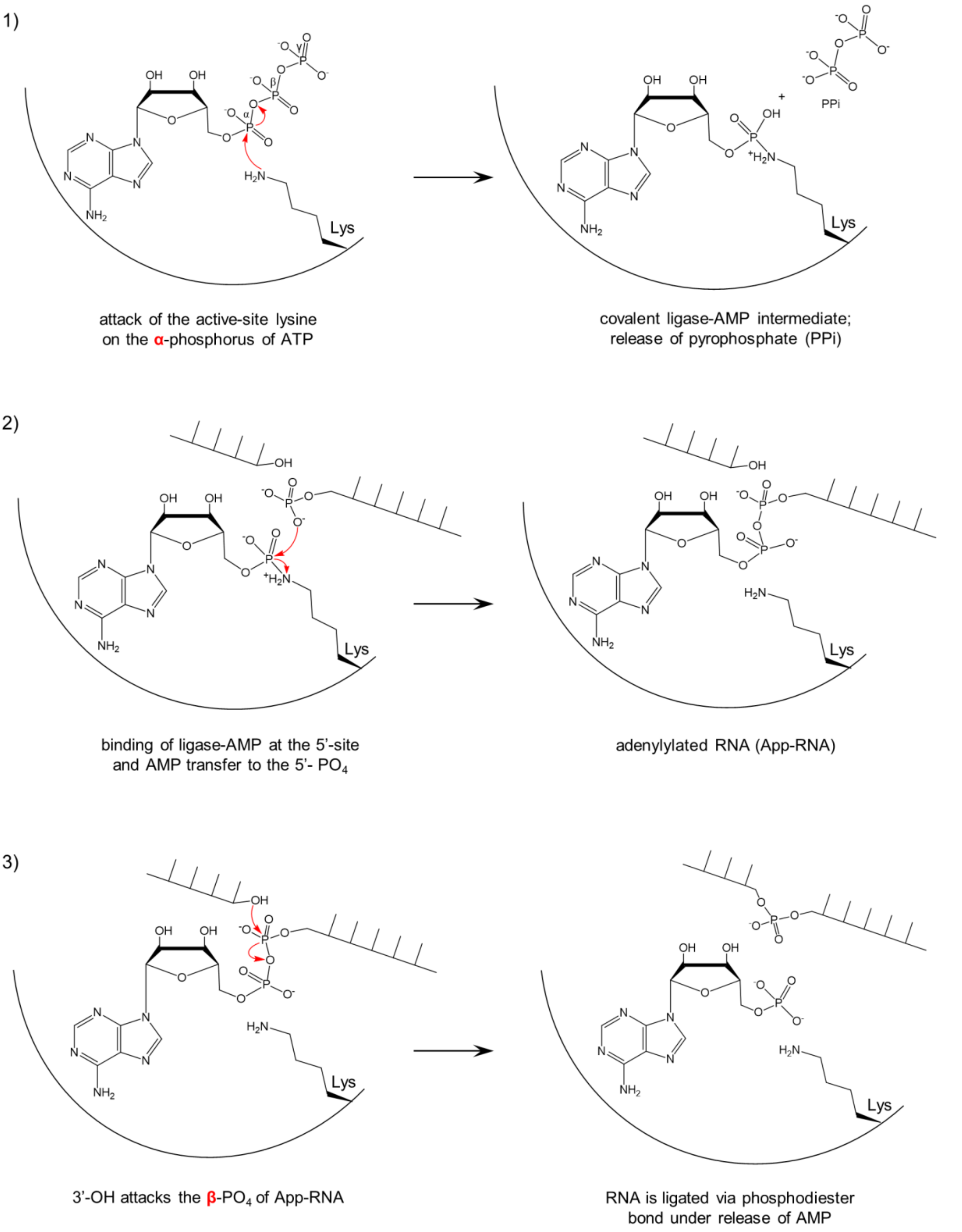

Supplementary Figure S7. Scheme of a classic ATP-dependent RNA ligation with T4 RNA ligase 1.
Classic ATP dependent RNA ligation mediated by an activated lysine in the active site of a T4 ligase.

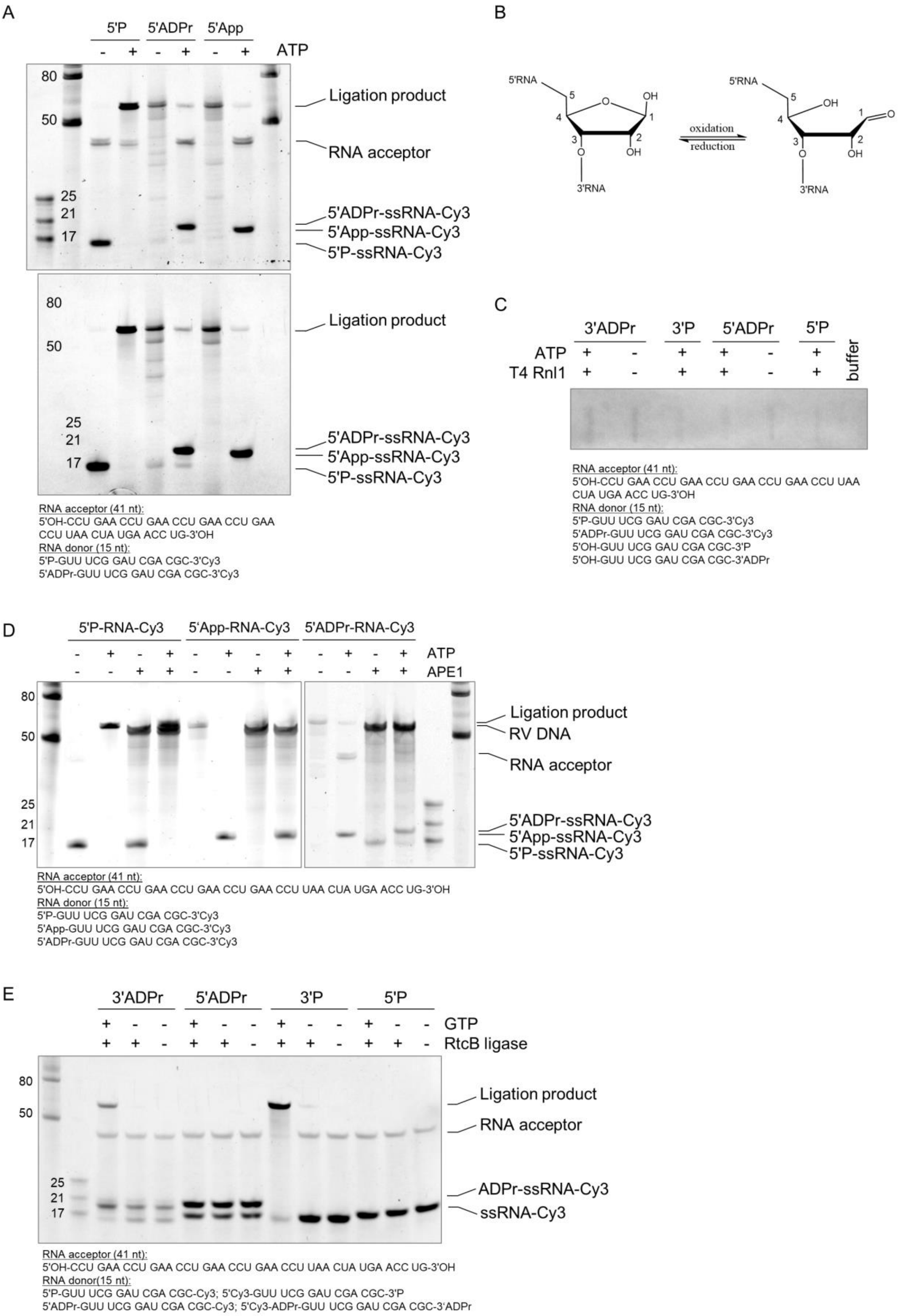

Supplementary Figure S8. Ligation products of 5'ADPr-RNA comprises an abasic site.
(A) A 15 nt 5'-phosphorylated and 3'-Cy3 labelled synthetic ssRNA oligo was adenylylated (App-RNA), using the 5' DNA Adenylation Kit (NEB), or ADP-ribosylated. After purification, 0.8 µM of 5'-monophopshorylated RNA, 5'-App-RNA or 5'-ADPr-RNA were used in a ligation assay with 2 µM RNA

acceptor (41 nt) and 20 U T4 Rnl1. Reactions were resolved via urea-PAGE after proteinase K treatment. In-gel fluorescence was detected (lower panel) and gel was stained with SYBR gold nucleic acid gel stain (upper panel). **(B)** Scheme of an abasic site in closed/reduced (left) and open/oxidised (right) conformation. **(C)** 5'- and 3'-ADP-ribosylated RNA oligos were ADP-ribosylated as described in (A). Modified and non-modified RNA-donors were ligated with 2 µM acceptor RNA oligo by 10 U T4 Rnl1 ligase in presence and absence of 1 mM ATP at 16°C for 16 h. T4 Rnl1 was heat deactivated at 65°C for 15 min. Ligation reactions were incubated with a biotin-labelled aldehyde-reactive probe, ARP, at 37°C for 1 h followed by cross-linking with PFA and RNA purification. Samples were blotted onto a positively charged nylon membrane and probed for abasic sites via immunostaining using Streptavidin-HRP (ab7403). **(D)** The urea-gel of **Figure 4E** was dyed with SYBR gold nucleic acid gel stain to visualise the RNA ligation acceptor as well as the reverse complement DNA oligo that was needed for APE1 cleavage**. (E)** 5'-phosphorylated and 3'-Cy3 labelled, or 3'-phosphorylated and 5'-Cy3 labelled synthetic ssRNA oligos were ADP-ribosylated with TRPT1 or PARP10cat, respectively, at 37ºC for 30 min followed by purification. 0.5 µM of 5'- and 3'-phosphorylated oligos, ~0.5 µM of 5'-ADP-ribosylated (~70% modification rate; ~0.35 µM ADPr-RNA) and ~0.15 µM of 3'-ADP-ribosylated (~65% modification rate; ~0.1 µM ADPr-RNA) RNA oligo were used in a ligation assay (+/-0.1 mM GTP; 37°C 1 h) with 1.75 µM of a non-phosphorylated RNA oligo as RNA-acceptor and 30 pmol RtcB ligase. Reactions were resolved via urea-PAGE after proteinase K treatment. Gels were stained with SYBR gold nucleic acid gel stain.

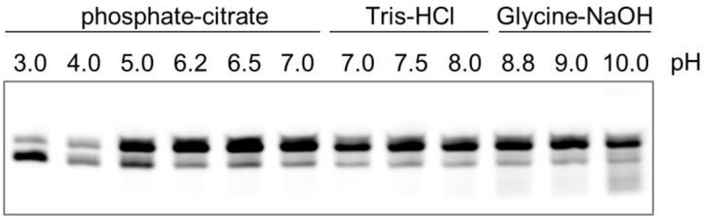

Supplementary Figure S9. ADP-ribosylation of RNA is acid-labile.
A 5'-phosphorylated ssRNA oligo (5'P-GUU UCG GAU CGA CGC-Cy3) was ADP-ribosylated with TRPT1 and subsequently purified. The resulting ADPr-RNA was incubated for 2 h at RT with the displayed buffers covering a pH range from 3.0–10.0.

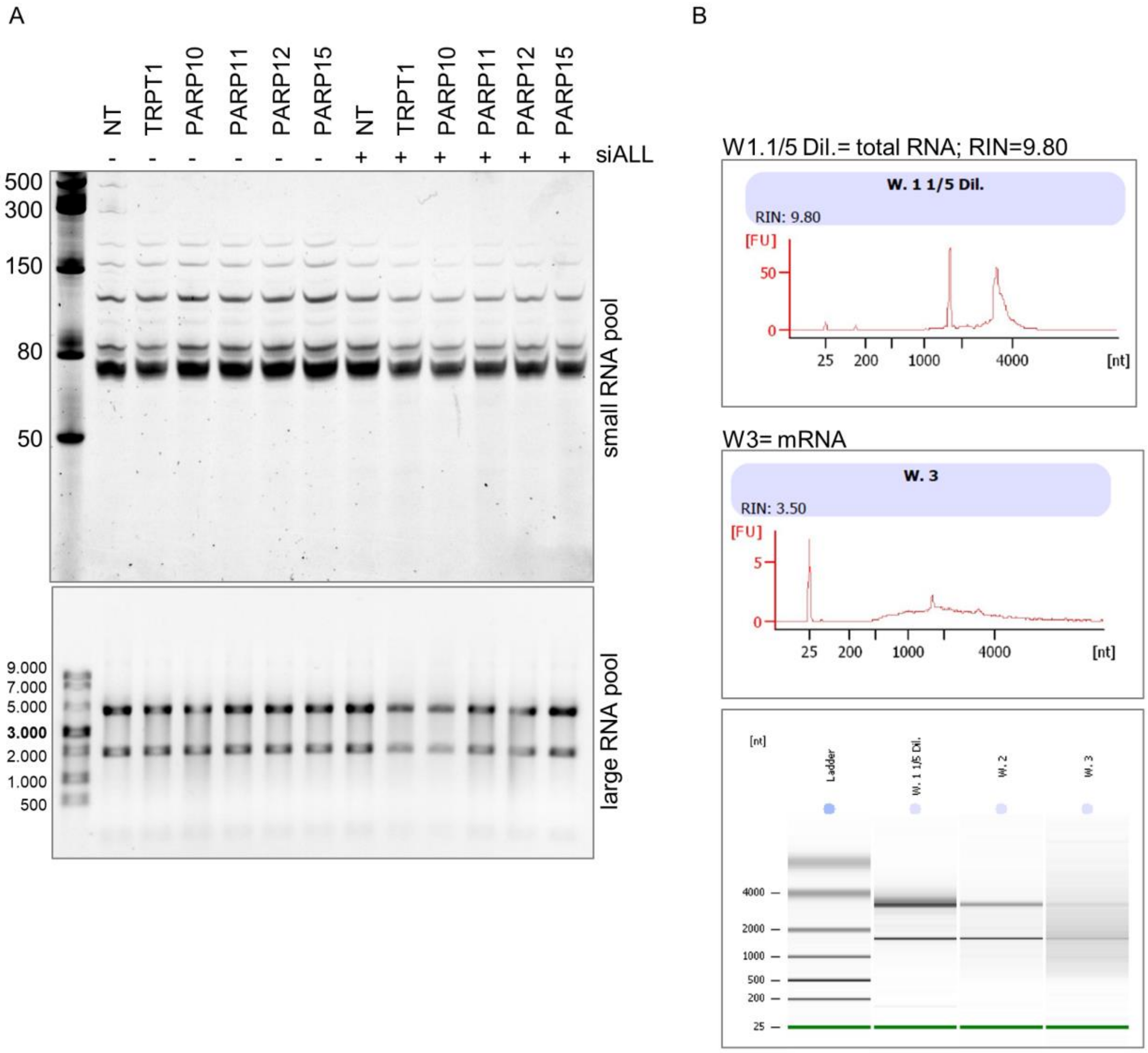

Supplementary Figure S10. Isolated RNA pools have high quality and optimal integrity.
(A) HeLa cells were reverse transfected with siRNA to knock-down TARG1, PARG and ARH3. 48 h later indicated PARPs were transiently transfected. After 24 h, cells were harvested and total, large, and small RNA pools were isolated. mRNA was enriched from the total RNA fraction. The small RNA pool was resolved on a denaturing urea-gel. The large RNA pool was separated on a 1% agarose gel. RNA was stained with SYBR gold nucleic acid gel stain.
(B) HeLa cells were prepared as in described in (A). Total and mRNA was analysed via high-resolution electrophoresis on the Agilent 2100 Bioanalyzer system. The RNA integrity number (RIN) for total RNA was defined to be 9.8. Integrity of total RNA and enrichment of mRNA are additionally represented as electropherograms.

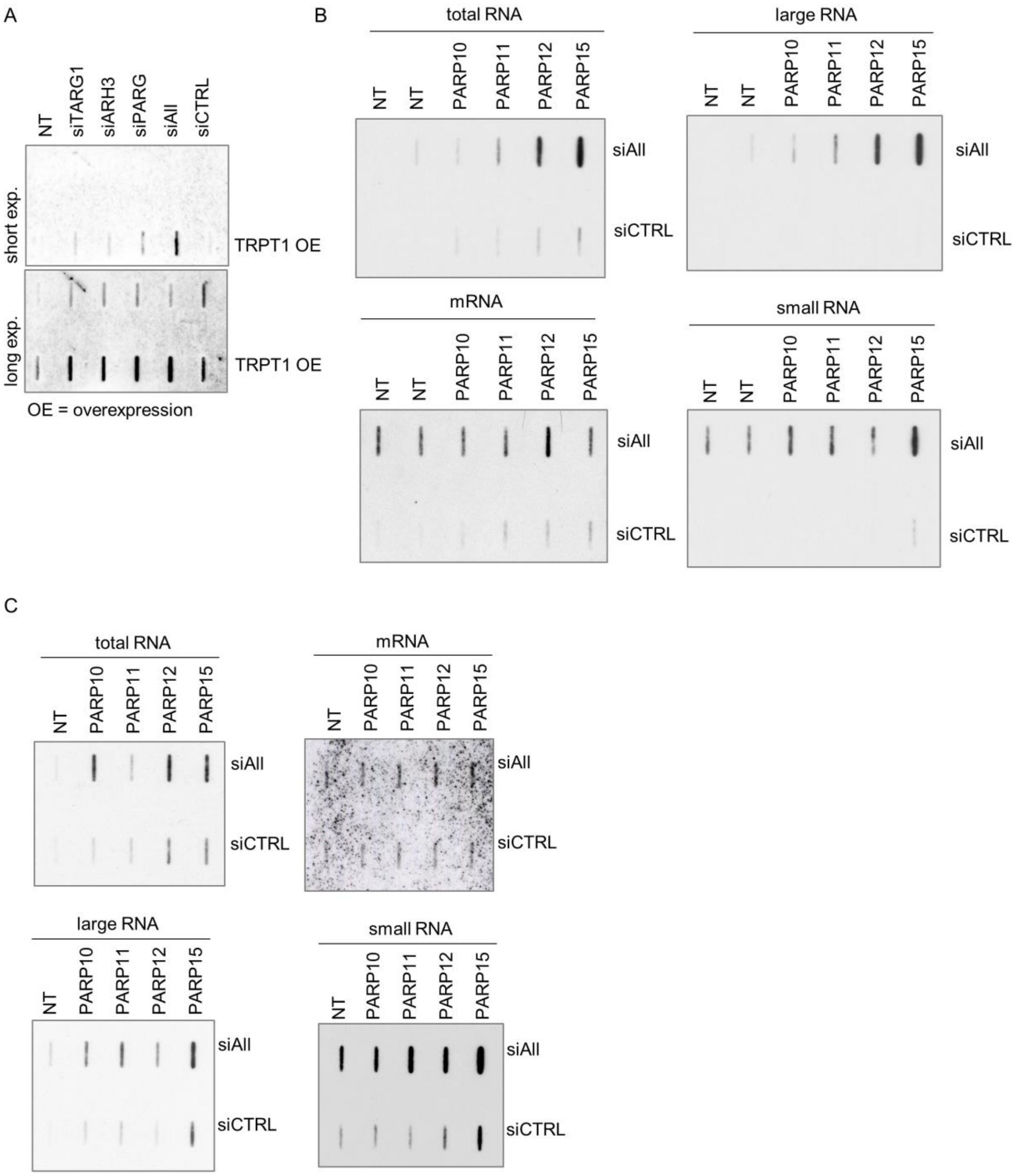

Supplementary Figure S11. Several mono-ARTs increase mRNA ADP-ribosylation upon overexpression.

(A) Replicate of the blot from Figure 1B. **(B)** Non cut blots form Figure 6A-D. **(C)** Replicates for Figure 6A-D. HeLa cells were reverse transfected with siRNA to knock-down TARG1, PARG and ARH3. 48 h later, indicated PARPs were transiently transfected. After 24 h cells were harvested and total, large, and small RNA pools were isolated. mRNA was enriched from the total RNA fraction. Samples were blotted onto nylon membrane and probed via immunoblotting using the PAR/MAR antibody (CST). NT= non-transfected.

Supplementary table S1. Oligonucleotides used in this study.

Name	Length (nt)	Sequence (5'→3')
5'P-ssRNA-Cy3	15	[Phos] GUU UCG GAU CGA CGC [Cy3]
5'P-G-ssRNA	15	[Phos] GUU UCG GAU CGA CGC
5'P-C-ssRNA	15	[Phos] CUU UCG GAU CGA CGC
5'P-A-ssRNA	15	[Phos] AUU UCG GAU CGA CGC
5'P-U-ssRNA	15	[Phos] UUU UCG GAU CGA CGC
5'Cy3-ssRNA-3'P	15	[Cy3] GUU UCG GAU CGA CGC [Phos]
RNA acceptor	41	CCU GAA CCU GAA CCU GAA CCU GAA CCU UAA CUA UGA ACC UG
IVT product_27 nt	27	[tri-phos] GGG CCA AGU UUC GGA UCG ACG CUC GUC
IVT product_40 nt	40	[tri-phos] AGG CCU CUC GCU CUG CUG GGU GUG CGC UUG CUU GGC UUG C
*G*Luc reporter	629	CAGTAATACGACTCACTATAGGGAGGACTCACTATTT GTTTTCGCGCCCAGTTGCAAAAAGTGTCGCCACCAT GGGAGTCAAAGTTCTGTTTGCCCTGATCTGCATCGCT GTGGCCGAGGCCAAGCCCACCGAGAACAACGAAGA CTTCAACATCGTGGCCGTGGCCAGCAACTTCGCGAC CACGGATCTCGATGCTGACCGCGGGAAGTTGCCCG GCAAGAAGCTGCCGCTGGAGGTGCTCAAAGAGATG GAAGCCAATGCCCGGAAAGCTGGCTGCACCAGGGG CTGTCTGATCTGCCTGTCCCACATCAAGTGCACGCC CAAGATGAAGAAGTTCATCCCAGGACGCTGCCACAC CTACGAAGGCGACAAAGAGTCCGCACAGGGCGGCA TAGGCGAGGCGATCGTCGACATTCCTGAGATTCCTG GGTTCAAGGACTTGGAGCCCATGGAGCAGTTCATCG CACAGGTCGATCTGTGTGTGGACTGCACAACTGGCT GCCTCAAAGGGCTTGCCAACGTGCAGTGTTCTGACC TGCTCAAGAAGTGGCTGCCGCAACGCTGTGCGACCT TTGCCAGCAAGATCCAGGGCCAGGTGGACAAGATCA AGGGGGCCGGTGGTGACTAAGAGAGCTCGCTTTCTT GCTG
reverse complementary DNA	57	GCG TCG ATC CGA AAC TCA GGT TCA TAG TTA AGG TTC AGG TTC AGG TTC AGG TTC AGG

Supplementary table S2. Plasmids used for recombinant protein expression

**Protein construct**	**Tag**	**Expression host**	**Plasmid (Vector/Insert)**
hMACROD1	His	BL21-CodonPlus (DE3)-RIL	pNH-TrxT-MACROD1-58–325
hMACROD1_G270E	His	BL21-CodonPlus (DE3)-RIL	pNH-TrxT-MACROD1-58–325-G270E
hTARG1	His	BL21-CodonPlus (DE3)-RIL	pDEST17-TARG1
hTARG1_G123E	His	BL21-CodonPlus (DE3)-RIL	pDEST17-TARG1
hPARGcat	His	Lemo21(DE3) E. coli	pNH-TrxT-PARGcat-448–976
hDXO	His	Rosetta™(DE3)pLysS	pDEST17-DXO
hDXO_E253A	His	Rosetta™(DE3)pLysS	pDEST17-DXO-E253A
hARH1	His	BL21-CodonPlus (DE3)-RIL	pNIC-Bsa4-ARH1
hARH2	His	BL21-CodonPlus (DE3)-RIL	pDEST17-ARH2
hARH3	His	BL21-CodonPlus (DE3)-RIL	pDEST17-ARH3
hPARP10cat	GST	BL21-CodonPlus (DE3)-RIL	pGST-PARP10-818–1025
hPARP10	His	BL21-CodonPlus (DE3)-RIL	pDEST17-PARP10
hTRPT1	His	Rosetta™(DE3)pLysS	pDEST17-hTRPT1-iso1
hPARP11	His	Rosetta™(DE3)pLysS	pET28-PARP11
hPARP11cat	His	Rosetta™(DE3)pLysS	pET28-PARP11-121-331

Supplementary table S3. List of siRNA oligonucleotides used in this study.

Gene	Vendor	Cat. No.
Control oligo	Dharmacon	D-001206-14-20
ARH3 (smart pool)	Horizon Discovery	M-020822-01-0010
TARG1	Dharmacon	J-015886-18-0010
PARG (smart pool)	Dharmacon	M-011488-02
